# Coumarin-quinolinium based fluorescent probe for free thiol detection and cancer cell imaging

**DOI:** 10.1039/d6an00818f

**Published:** 2026-11-05

**Authors:** Mohammed Adnan Abdulsalam Abdulsalam, Halil Duyar, Ferda Hacivelioglu, Burak Derkus, Jonathan L. Sessler, Ergin Yalcin, Mehmet Gokhan Caglayan

**Affiliations:** a Ankara University, Faculty of Pharmacy, Department of Analytical Chemistry Ankara Turkiye gcaglayan@ankara.edu.tr; b Ankara University, Graduate School of Health Sciences Turkiye; c Department of Chemistry, Gebze Technical University Gebze Kocaeli Turkiye; d Ankara University, Department of Chemistry Ankara Turkiye; e Department of Chemistry, The University of Texas at Austin 105 East 24th Street-A5300 Austin Texas 78712-1224 USA sessler@cm.utexas.edu ergin.yalcin@iste.edu.tr; f Department of Engineering Basic Sciences, Faculty of Natural Sciences and Engineering, Iskenderun Technical University Hatay Turkiye

## Abstract

A coumarin-quinolinium hybrid fluorescent probe was designed and synthesized through a three-step sequence. The probe exhibited moderate emission at 497 nm when excited at 407 nm and demonstrated an increase in fluorescence intensity upon reaction with free thiols in aqueous media (*e.g.*, 5% DMSO in pH 7 Britton–Robinson buffer, v/v). Additionally, its effective cell-staining capability facilitated successful application in cell imaging. The probe was found to display enhanced fluorescence in cancerous lung cells (A549) and colon cells (HT-29) relative to healthy lung cells (BEAS-2B) and colon cells (FHC). The probe thus permitted discrimination of cancer cells from healthy cells, a finding consistent with interaction and reaction with glutathione. The analytical performance of the probe was further studied, and it was found to exhibit a linear response to cysteine and glutathione over a wide concentration range. Accuracy, precision, and sustained performance were evidenced by recovery rates close to 100% and low relative standard deviation (RSD) values under conditions of repeated use. The present probe also permitted detection of the presence and absence of free thiols in two representative proteins, human serum albumin and alpha-synuclein, respectively, where it was found to provide confirmatory support for their proposed tertiary structures.

## Abstract 2

A coumarin-quinolinium probe allows monitoring of protein tertiary structures and intracellular biothiol fluctuations, thus providing a reliable optical-based strategy for differentiating cancerous cells from healthy ones.

## Graphical abstract

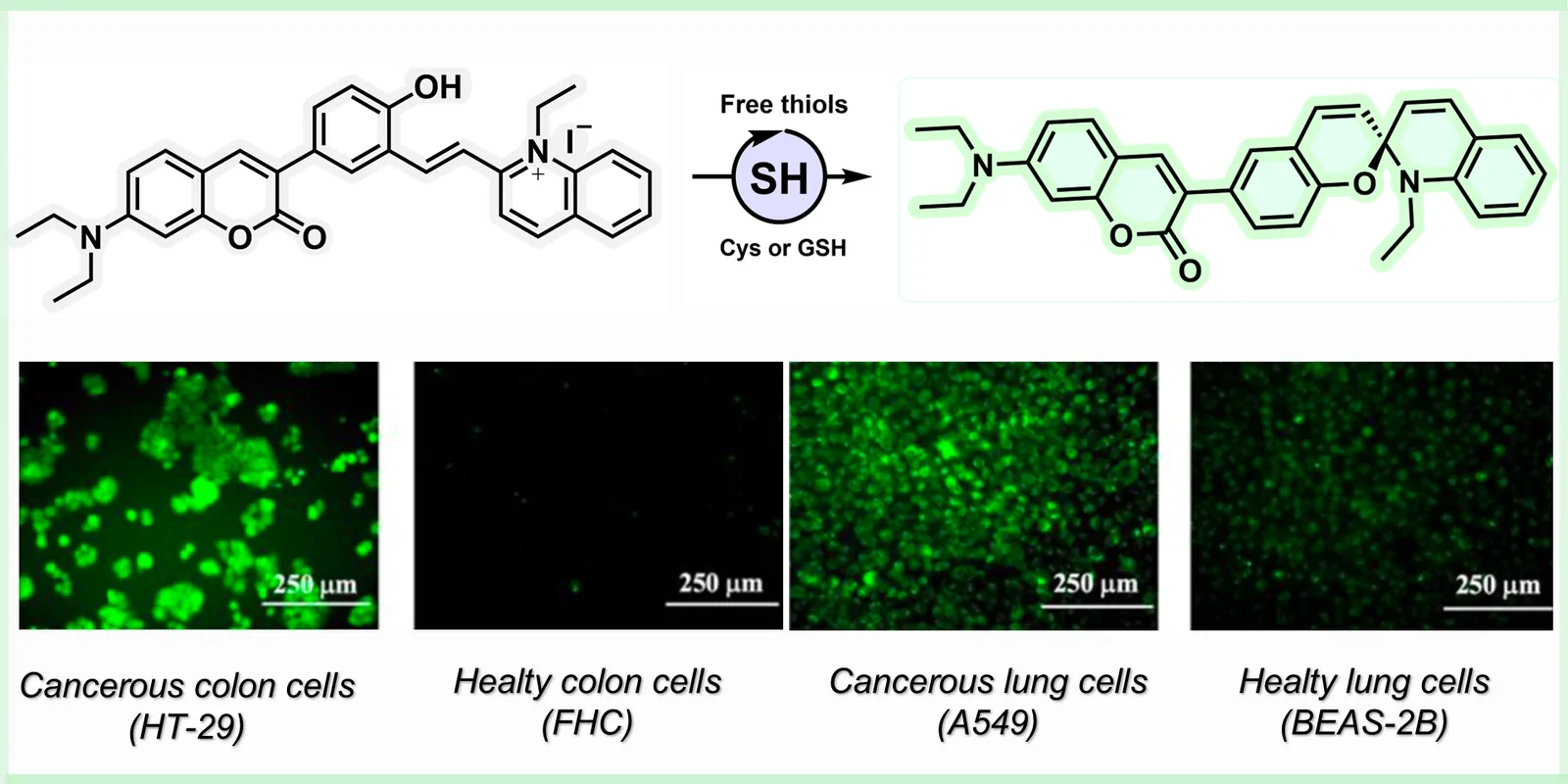

## 1. Introduction

Thiol-containing biomarkers, characterized by the presence of reactive sulfhydryl (–SH) groups, play pivotal roles in diverse biological processes, including redox signaling, enzymatic catalysis, and the structural stabilization of proteins.^1–3^ Thiols are highly sensitive to oxidative environments, undergoing reversible transformations into disulfide bonds, a transformation that can critically influence protein folding, activity, and stability.^4,5^ The dynamic equilibrium between free thiols and disulfides is essential for maintaining cellular homeostasis, while its dysregulation, particularly aberrant disulfide bond formation, contributes to protein misfolding, oxidative stress, and the onset of various pathological conditions, including neurodegenerative and metabolic disorders.^6,7^

A deeper understanding of these redox mechanisms requires the precise discrimination between native free thiols and disulfide bond. This is often achieved by comparative analyses across multiple labeling steps, which improve the accuracy of thiol profiling and yields more reliable insights into disease-related redox biology.^8,9^ In addition, thiol-containing metabolites, such as glutathione (GSH), are elevated in many tumor cells, enabling enhanced resistance to oxidative stress.^10^ This augmented GSH pool not only promotes cancer cell survival but can also underlie therapeutic resistance by neutralizing chemotherapeutic agents and supporting redox adaptation.^11,12^ Accordingly, the reliable determination and imaging of free thiol biomarkers could lead to improvements in disease diagnosis, prognosis monitoring, and provide insights into the expected therapeutic efficacy of new experimental agents.

Here we report the design and synthesis of a coumarin–quinolinium hybrid fluorescent molecule (P3) as a water-soluble probe for selective free thiol detection. As detailed below, upon interaction with thiol-containing molecules, P3 exhibits a pronounced fluorescence enhancement in the green emission region. It also displays aqueous solubility and a large Stokes shift (∼90 nm). The analytical performance of P3 was validated using cysteine (Cys) and glutathione (GSH), while its practical applicability was demonstrated through discrimination of cancer (HT-29 and A549) over healthy (FHC and BEAS-2B) cell lines. Moreover, P3 proved effective in monitoring free thiols in model proteins, highlighting its potential use in investigating protein structural characteristics and redox-related biological processes.

Several analytical techniques have been developed for free thiol detection including high-performance liquid chromatography,^13^ capillary electrophoresis,^14^ voltammetry,^15,16^ flow injection analysis,^17,18^ and mass spectrometry.^19^ While these methods, especially separation-based strategies, offer high analytical performance and are well suited for routine analyses, their widespread application is limited by the need for expensive instrumentation, labor-intensive workflows, and incompatibility with multiplexed or real-time measurements. In contrast, optical probes and sensors have emerged as promising alternatives, offering high-throughput, cost-effective, and user-friendly platforms that can be readily integrated into routine thiol determination and cellular imaging.

Among the various optical thiol sensing strategies pursued to date, fluorescence-based probes have emerged as particularly attractive owing to their high sensitivity, excellent spatial resolution, and applicability in living systems. Fluorescent imaging enables direct visualization of cellular processes and can provide valuable information regarding physiological and pathological changes at the cellular level.^20,21^ In cancer cells, increased oxidative stress results in elevated intracellular levels of antioxidant thiol-containing molecules, particularly GSH, which has encouraged the development of fluorescence-based approaches for distinguishing malignant and normal cells.^22,23^ An ideal fluorescent thiol probe should exhibit a significant fluorescence enhancement upon target interaction, long-wavelength emission, a large Stokes shift to minimize spectral overlap, and good water compatibility for biological applications.

Various fluorescent probes have been developed for thiol detection based on different mechanisms, including aromatic substitution rearrangements, aldehyde cyclization, Michael addition, Se–N or S–S bond cleavage, acrylate addition-cyclization, metal coordination, and nucleophilic substitution reactions, among others.^24,25^ Although collectively these strategies have led to valuable advances, the development of probes combining high selectivity, strong fluorescence enhancement, aqueous compatibility, and applicability in complex biological systems remains an important challenge.

Among reported fluorophore platforms, coumarin derivatives have received considerable attention due to their excellent photophysical properties, including high quantum yields, tuneable optical characteristics, and large Stokes shifts, making them attractive candidates for biological sensing applications.^26,27^ This promise led us to consider that probe P3 might have a role to play in the detection of biologically important thiols. The present study was undertaken as a test of this proposition. As detailed below, probe P3 was indeed found to be effective as thiol-detection system. In particular, it allowed differentiation between cancerous and non-cancerous cells *in vitro*, as well as characterization of food supplement and other thiol-containing entities.

## 2. Materials and methods

### 2.1. Reagents, chemicals and instruments

Unless otherwise specified, all reagents were purchased from commercial suppliers and used without further purification. Solvents were purified using conventional methods prior to use. Methanol (MeOH) was purchased from Sigma-Aldrich, while all other chemicals used for molecular synthesis including 4-hydroxyphenylacetic acid, 4-diethylamino-2-hydroxybenzaldehyde, triethylamine, acetic anhydride, dichloromethane (CH_2_Cl_2_), acetic acid (AcOH), hexamethylenetetramine (HMTA), deionized water, 1-ethyl-2-methylquinolinium iodide, and anhydrous ethanol were procured from BLD Pharm. Phosphoric acid (H_3_PO_4_, Sigma-Aldrich), acetic acid (CH_3_COOH, Sigma-Aldrich), boric acid (H_3_BO_3_, Sigma-Aldrich) Milli-Q ultrapure water (Millipore, USA), and sodium hydroxide (NaOH, Sigma-Aldrich) were used to prepare the Britton–Robinson buffer. The following reagents were used at various stages of the study: magnesium sulfate (MgSO_4_, Sigma-Aldrich), zinc sulfate (ZnSO_4_, Macklin), thiamine (vitamin B1, Macklin), folic acid (Macklin), coumarin 6 (BLD), glycine (BLD), l-lysine (BLD), glutathione (GSH, BLD), l-carnitine (BLD), l-arginine (Macklin), l-cysteine (l-Cys, Sigma-Aldrich), d-cysteine (d-Cys, Sigma-Aldrich), homocysteine (Hcy, Sigma-Aldrich), sodium borohydride (NaBH_4_, Sigma-Aldrich), bovine serum albumin (BSA, Sigma-Aldrich), α-synuclein (AlexoTech), α-lipoic acid (ALA, Sigma-Aldrich), acetonitrile (ACN, Sigma-Aldrich), dimethyl sulfoxide (DMSO, C_2_H_6_SO, Sigma-Aldrich), dimethylformamide (DMF, Sigma-Aldrich), and commercial food supplement capsules containing biotin, l-cysteine and zinc. DMSO-*d*_6_ was obtained from Cambridge Isotope Laboratories. All other chemicals were obtained from Sigma-Aldrich or Merck and used as received.

All stock solutions were prepared fresh, sonicated for 10 minutes in an ultrasonic bath (J.P. Selecta Co.), protected from light with aluminum foil, and stored at 4 °C.

Spectroscopic measurements were performed using a Cary Eclipse fluorescence spectrophotometer (Agilent Technologies, Santa Clara, USA) and a Cary 60 UV-Vis spectrophotometer (Agilent Technologies, Santa Clara, USA). Fluorescence microscopy was carried out on a Zeiss (Axiovert 5 digital microscope) inverted fluorescence microscope. FT-IR spectra were recorded on a Bruker Alpha-P in ATR in the range of 4000–650 cm^−1^. Nuclear magnetic resonance (NMR) spectroscopy was performed using a Q.one Instruments Quantum I^PLUS^ 500 MHz NMR spectrometer and Varian Mercury 400 instrument. Mass spectrometric studies were carried out using a Bruker MALDI-TOF (matrix-assisted laser desorption/ionization-time-of-flight) system or Agilent 6120 instruments.

Human cancer cell lines HT-29 (colorectal adenocarcinoma, ATCC HTB-38) and A549 (lung carcinoma, ATCC CCL-185), as well as healthy cell lines FHC (normal human colon, ATCC CRL-1831) and BEAS-2B (normal human bronchial epithelium, ATCC CRL-3588), were purchased commercially and used for the cellular imaging studies.

### 2.2. Synthesis of P3

#### 2.2.1. Synthesis of compound P1

Compound P1 was synthesized from 4-diethylamino-2-hydroxybenzaldehyde and 4-hydroxyphenylacetic acid using a reported procedure with minor modifications.^28,29^ Briefly, 4-hydroxyphenylacetic acid (4.2 mmol, 0.639 g), 4-diethylamino-2-hydroxybenzaldehyde (4.0 mmol, 0.772 g), triethylamine (40 mmol, 1.05 mL), and acetic anhydride (19 mmol, 1.8 mL) were mixed in a microwave reaction tube and irradiated at 140 °C for 20 min. After cooling to room temperature, the crude mixture was purified by column chromatography over silica gel (CH_2_Cl_2_, eluent) and recrystallized from ethanol to afford P1 as a yellow solid (0.913 g, 2.6 mmol, 65% yield).

4-(7-(Diethylamino)-2-oxo-2*H*-chromen-3-yl)phenyl acetate (compound P1): ^1^H NMR (500 MHz, DMSO-*d*_6_) *δ* 8.06 (s, 1H), 7.71 (d, *J* = 8.4 Hz, 2H), 7.47 (d, *J* = 8.8 Hz, 1H), 7.15 (d, *J* = 8.4 Hz, 2H), 6.71 (d, *J* = 8.8 Hz, 1H), 6.54 (s, 1H), 3.42 (q, *J* = 6.6 Hz, 4H), 2.27 (s, 3H), 1.11 (t, *J* = 6.8 Hz, 6H). ^13^C NMR (125 MHz, DMSO-*d*_6_) *δ* 169.3, 160.6, 155.9, 150.5, 149.8, 141.3, 133.2, 129.7, 129.1, 118.0, 109.2, 108.4, 96.1, 44.2, 20.9, 12.4 ppm; HRMS (MALDI) *m*/*z* calcd for C_21_H_21_NO_4_ [M + Na]^+^: 374.1363, found 374.1357 [Fig. S1a–c].

#### 2.2.2. Synthesis of P2

Compound P1 (0.351 g, 1.0 mmol) was dissolved in AcOH (10 mL) and cooled to 0 °C in an ice bath. HMTA (0.210 g, 1.5 mmol) was added in one portion, and the reaction mixture was heated under reflux with monitoring by TLC. Once the reaction was deemed complete, the mixture was cooled, diluted with water (50 mL), and the precipitate was collected by filtration. The crude solid obtained in this way was purified by silica gel column chromatography (CH_2_Cl_2_/MeOH = 100 : 1) to afford compound P2 as a yellow solid (118 mg, 0.35 mmol, 35% yield). For 5-(7-(diethylamino)-2-oxo-2*H*-chromen-3-yl)-2-hydroxybenzaldehyde (P2): ^1^H NMR (400 MHz, DMSO-*d*_6_) *δ* 10.87 (s, 1H), 10.31 (s, 1H), 8.07 (s, 1H), 8.03 (s, 1H), 7.87 (d, *J* = 8.7 Hz, 1H), 7.51 (d, *J* = 8.6 Hz, 1H), 7.05 (d, *J* = 8.7 Hz, 1H), 6.73 (d, *J* = 8.7 Hz, 1H), 6.56 (s, 1H), 3.44 (q, *J* = 6.8 Hz, 4H), 1.13 (t, *J* = 6.8 Hz, 6H). ^13^C NMR (100 MHz, DMSO-*d*_6_) *δ* 191.2, 160.6, 160.2, 155.7, 150.4, 140.3, 135.9, 129.6, 128.3, 126.9, 122.0, 117.6, 117.1, 109.2, 108.5, 96.2, 44.1, 12.4 ppm; HRMS (MALDI): *m*/*z* calcd for [M + Na]^+^ C_20_H_19_NO_4_ = 360.1206; found = 360.1210 [Fig. S2a–c].

#### 2.2.3. Synthesis of P3

A mixture of 1-ethyl-2-methylquinolinium iodide (300 mg, 1.0 mmol) and compound P2 (337 mg, 1.0 mmol) in anhydrous ethanol (10 mL) containing a catalytic amount of piperidine was heated at reflux for a day. The mixture was cooled to room temperature, concentrated under reduced pressure, and the crude product purified by silica gel column chromatography (CH_2_Cl_2_/MeOH = 250 : 1, eluent) to give P3 as a dark green powder (92.7 mg, 0.15 mmol, 15% yield). For (*E*)-2-(5-(7-(diethylamino)-2-oxo-2*H*-chromen-3-yl)-2-hydroxystyryl)-1-ethylquinolin-1-ium iodide (P3): ^1^H NMR (500 MHz, DMSO-*d*_6_) *δ* 10.98 (s, 1H), 9.03 (d, *J* = 8.9 Hz, 1H), 8.57 (dd, *J* = 16.0, 9.0 Hz, 2H), 8.37 (d, *J* = 8.0 Hz, 1H), 8.29 (d, *J* = 15.7 Hz, 1H), 8.21–8.14 (m, 2H), 8.08–8.00 (m, 2H), 7.94 (t, *J* = 7.5 Hz, 1H), 7.73–7.67 (m, 1H), 7.50 (d, *J* = 8.9 Hz, 1H), 7.05 (d, *J* = 8.6 Hz, 1H), 6.73 (dd, *J* = 9.0, 1.8 Hz, 1H), 6.56 (d, *J* = 1.8 Hz, 1H), 5.08 (q, *J* = 7.1 Hz, 2H), 3.44 (q, *J* = 6.9 Hz, 4H), 1.59 (t, *J* = 7.2 Hz, 3H), 1.13 (t, *J* = 7.0 Hz, 6H). ^13^C NMR (125 MHz, DMSO-*d*_6_) *δ* 160.7, 157.3, 155.9, 155.7, 150.3, 144.4, 143.8, 140.3, 138.1, 135.2, 132.9, 130.5, 130.2, 129.5, 128.9, 128.1, 127.3, 121.4, 121.3, 118.9, 118.6, 118.3, 116.2, 109.2, 108.4, 96.2, 46.9, 44.1, 13.8, 12.4. (MALDI-TOF): *m*/*z* calcd for [M]^+^ C_32_H_31_N_2_O_3_ = 491.23; found = 490.129 ([M − H]^+^) [Fig. S3a–c].

### 2.3. Spectral measurements

A stock solution of P3 was prepared at a concentration of 10 µM in a mixture of DMSO 5% and 95% (v/v) Britton–Robinson buffer at pH 7. The absorption and fluorescence spectra were recorded by placing 3.0 mL of the solution into a quartz cuvette. The P3 stock solution proved stable for at least two months when stored at 4 °C, protected from light.

A stock solution of l-Cys and GSH was prepared at a concentration of 10 mM in 1.0 mL Britton–Robinson buffer (pH 7.0) and employed with the P3 solution for the titration studies.

Similarly, stock solutions of BSA, ALA, and α-*syn* were prepared under the same conditions and used for the corresponding experiments.

### 2.4. Imaging of cells

Human colorectal adenocarcinoma (HT29), normal colon epithelial (FHC), human lung adenocarcinoma (A549), and normal lung epithelial (BEAS-2B) cells were maintained under standard conditions (37 °C, 5% CO_2_) in their recommended growth media supplemented with 10% fetal bovine serum and 1% penicillin–streptomycin. For imaging experiments, cells were seeded on plastic-bottom 48-well plates and allowed to reach ∼70–80% confluence. Probe P3 was diluted freshly in culture medium and applied at final concentrations of 1, 2.5, 5, 7.5, and 10 µM; an untreated group with 0.1% DMSO served as negative control. 10 minutes after treating the cells with P3, the cells were examined on an inverted fluorescence microscope (Zeiss Axiovert 5 digital microscope, green channel: excitation ∼488 nm, emission ∼510–550 nm). For each condition and cell line, images were acquired using 5×, 10×, and 20× objectives under an identical exposure time, gain, and illumination intensity across the groups. Brightfield images were recorded in parallel for morphological reference. Confocal fluorescence images were acquired on a Zeiss LSM900 laser-scanning confocal microscope using [10× and 20×] objectives, with excitation at 488 nm and emission collected over 516 nm. HT-29 cells were prepared and stained as described above (2.5 µM P3, 10 min incubation).

## 3. Results and discussion

### 3.1. Synthesis and characterization of P3

The synthesis for P3 is outlined in Scheme 1. Starting from 4-(diethylamino)salicylaldehyde and *p*-hydroxyphenylacetic acid, compound P1 was obtained *via* a Perkin reaction. Subsequent Duff formylation of P1 using hexamethylenetetramine (HMTA) in acetic acid afforded the key aldehyde intermediate P2.^30^ Finally, P3 was synthesized through a condensation reaction between P2 and 1-ethyl-2-methylquinolinium iodide in ethanol under alkaline conditions.

**Scheme 1:**
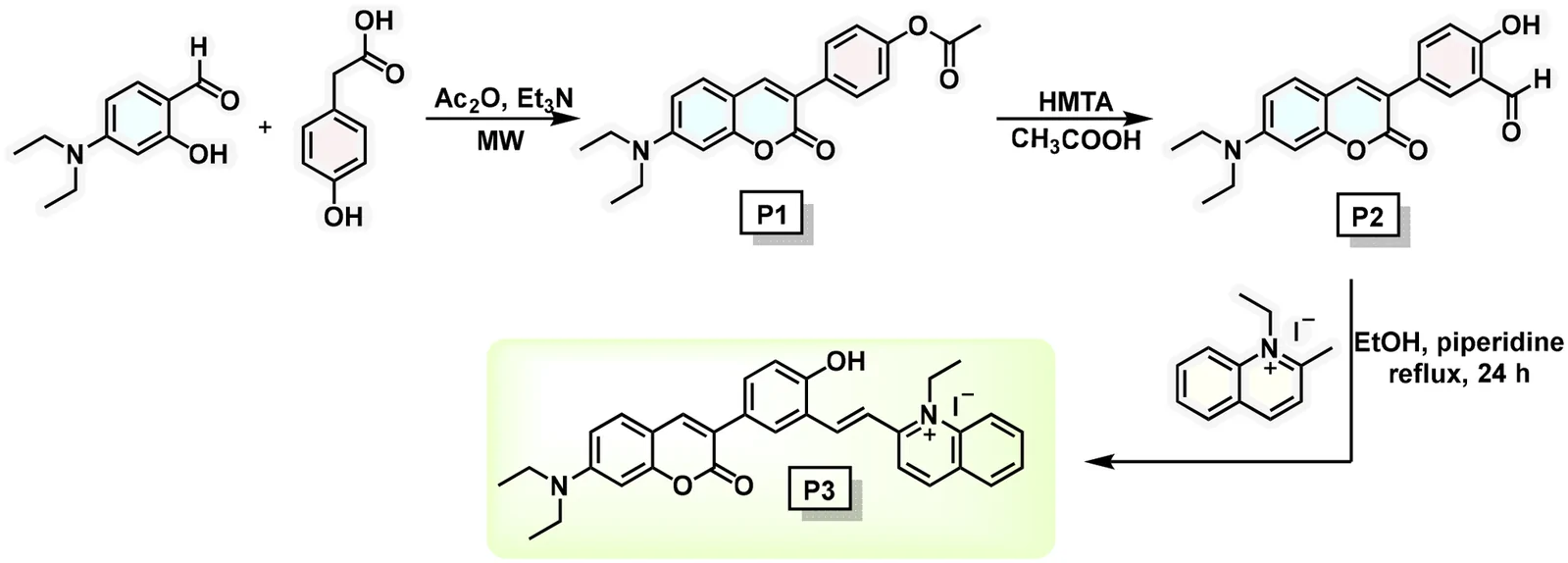
Synthetic pathway used to prepare probe P3.

The structures of all new compounds were confirmed by spectroscopic methods. For P3, the ^1^H NMR spectrum (Fig. S3a) revealed characteristic resonances in both the aromatic and aliphatic regions, while the ^13^C NMR spectrum (Fig. S3b) contained signals corresponding to the carbonyl, aromatic, and aliphatic carbons. High-resolution mass spectrometry (HRMS) analysis (Fig. S3c) confirmed the molecular formula, with excellent agreement being seen between the calculated and observed values.

The optical properties of P3 were investigated using UV-vis and fluorescence spectroscopies. It was found to exhibit a strong absorption band at 407 nm, which is attributed to a π–π* transition (*cf.* Fig. S4a and b). Upon excitation at this wavelength, a prominent fluorescence emission centered at 497 nm is seen. This corresponds to a Stokes shift of ∼90 nm. This relatively large Stokes shift is considered advantageous for fluorescence sensing and imaging applications, as it reduces self-absorption and improves signal resolution. The fluorescence quantum yield of P3 was determined relative to coumarin 153 (*Φ* = 54% in ethanol^31^), giving *Φ* = 3.3% in DMSO and 1.0% in 5% DMSO in water (v/v). The lower value in the aqueous medium supports the intramolecular charge-transfer character proposed for P3 in that non-radiative deactivation proved more efficient in polar, protic media.

### 3.2. Solvent effects on the fluorescence features of P3

In solution, the fluorescence properties of P3 were found to be influenced strongly by the polarity of the solvent and its hydrogen-bonding ability. In relatively less polar solvents, such as ethanol, DMF, and acetonitrile, P3 exhibited an emission maximum at 475 nm. In contrast, in the highly polar aprotic medium, DMSO, the emission maximum shifted slightly to 480 nm. In a mixed aqueous medium (5% DMSO in water, v/v), the emission band underwent a more pronounced bathochromic shift to 497 nm (Fig. S5). This latter red shift is attributed to the stabilization of the excited state of P3 in polar and hydrogen-bonding environments, which lowers the energy gap between the ground and excited states. Such behavior is consistent with the observed solvatochromism and is thought to reflect the intramolecular charge-transfer (ICT) character of the probe. The solvatochromic response of P3 highlights its sensitivity to the microenvironment. This, in turn, led us to consider its potential applicability in probing polarity and hydrogen-bonding variations in biological systems.

### 3.3. Thiol-induced structural transformation of P3

The ability of probe P3 sense biothiols is based on a proposed analyte-induced intramolecular cyclization as shown in Fig. 1. P3 possesses a merocyanine-like conjugated structure containing a phenolic hydroxyl group positioned adjacent to a quinolinium moiety. The positively charged quinolinium unit, together with the extended π-conjugated system, increases the acidity of the phenolic proton and facilitates the formation of a nucleophilic phenoxide intermediate upon interaction with thiol-containing analytes. The sensing process is then initiated through an acid–base interaction in which the thiolate form of l-cysteine (l-Cys), by way of illustration, acts as a Brønsted base and abstracts the phenolic proton of P3. The resulting phenoxide intermediate then undergoes intramolecular nucleophilic attack at the electrophilic carbon center adjacent to the quaternary quinolinium moiety. This cyclization process results in the formation of the corresponding spirocyclic structure (spiro-P3). The conversion from the extended conjugated merocyanine structure to the spirocyclic form disrupts the π-conjugation pathway and alters the electronic distribution within the molecule, leading to the observed spectroscopic changes.

**Fig. 1:**
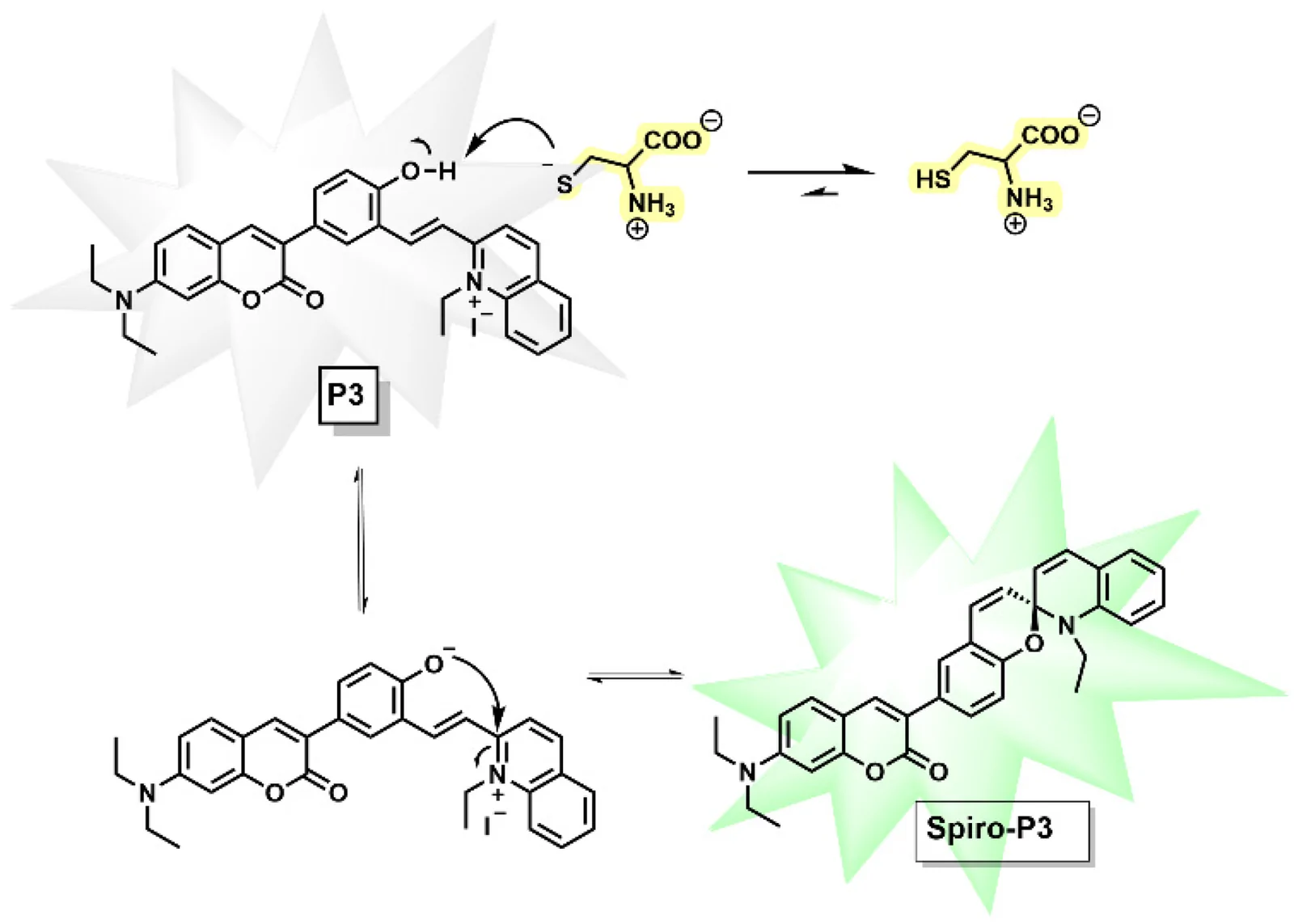
Proposed thiol-induced merocyanine-to-spirocyclic transformation mechanism for P3 using l-cysteine as a representative biothiol.

This proposed mechanism is consistent with the well-established merocyanine-to-spirocyclic transformation observed in related systems, where intramolecular ring closure is promoted by the generation of a phenoxide intermediate under appropriate environmental conditions, including changes in pH, solvent polarity, and analyte interactions.^32–34^

To verify experimentally this proposed mechanism, the interaction between P3 and biothiols was further investigated using NMR spectroscopy. In the specific case of l-Cys, titration into a solution of P3 in DMSO-*d*_6_, the resonance corresponding to the phenolic hydroxyl proton at 10.3 ppm disappeared, while new aromatic signals (H_a_–H_d_) emerged in the 6.80–7.55 ppm spectral region (Fig. S6). A new resonance at 3.1 ppm, assigned to the N–CH_2_ protons of spiro-P3 was also seen. In addition, the HMBC spectrum (Fig. S7) revealed correlations between the N–CH_2_ proton signals and the H_d_ proton resonance of the same carbon atom. Considered in concert, these data provide support for the conclusion that a free –SH functionality is essential for triggering the structural transformation of P3 and that the observed optical changes arise from conversion of P3 to spiro-P3.

The solvent-dependent behavior of the P3/spiro-P3 transformation was further investigated by NMR spectroscopy. In a DMSO-*d*_6_/H_2_O mixture (7 : 3, v/v), the proton resonances appeared as pairs of signals, which can be attributed to the presence of *E*/*Z* isomeric forms. The phenolic hydroxyl proton resonance, which disappeared after addition of l-Cys in pure DMSO-*d*_6_, was recovered in the presence of water (Fig. 2). The disappearance and subsequent recovery of the phenolic proton resonance in different solvent environments is taken as evidence that the P3/spiro-P3 transformation constitutes a reversible equilibrium process, rather than an irreversible chemical reaction. The conversion from the extended conjugated merocyanine structure to the spirocyclic form disrupts the π-conjugation pathway and removes the positive charge from the quinolinium nitrogen. Two spectroscopic consequences follow. First, the molar absorptivity at 407 nm decreases, since this band is associated with the conjugated form. Second, the rigid spirocyclic framework restricts torsional motion about the conjugated bridge; this restricts a non-radiative decay channel with the consequence that the emissive efficiency increases.

**Fig. 2:**
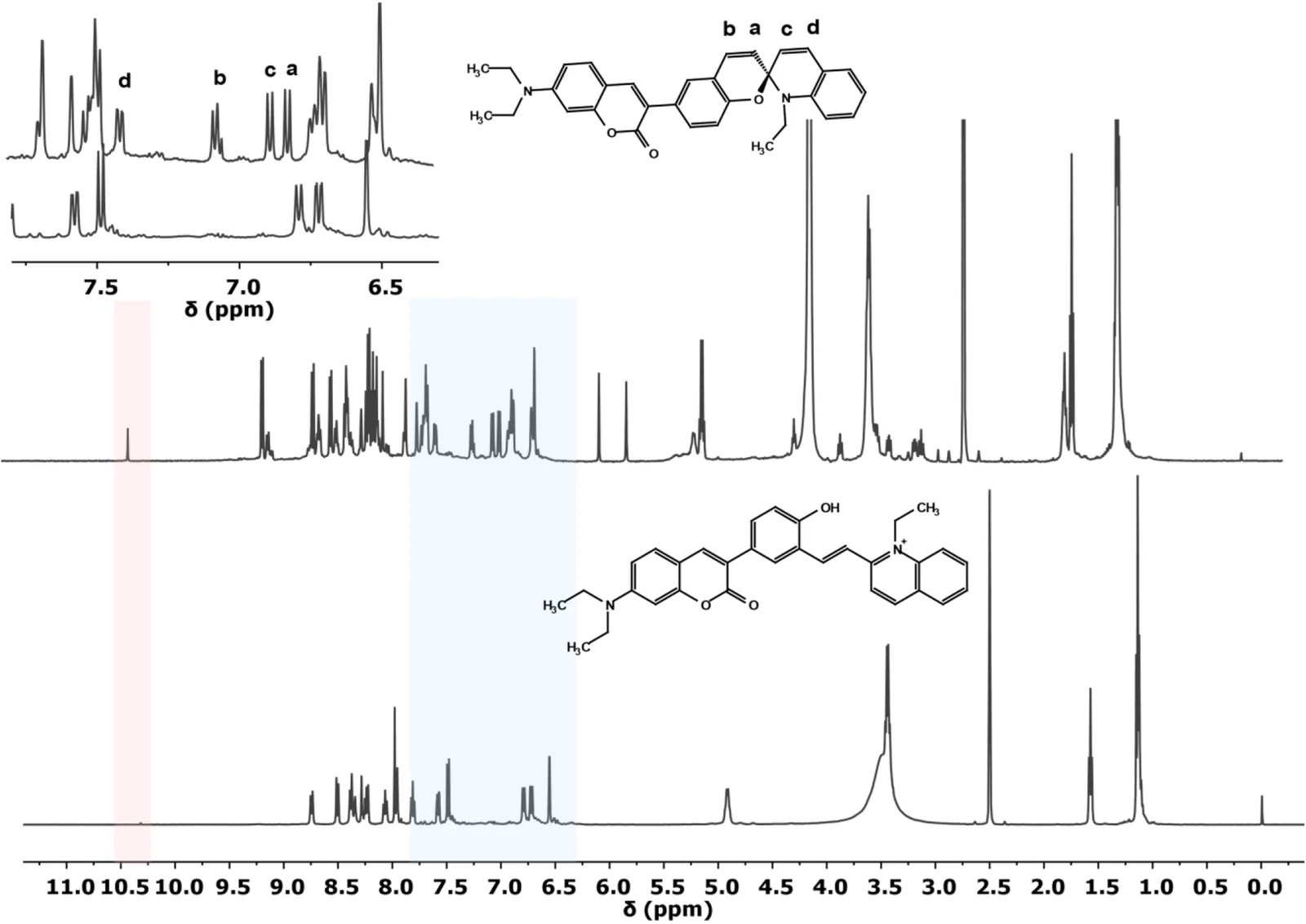
^1^H NMR spectra of P3 recorded in the absence and presence of l-Cys (3 eq.) in DMSO–H_2_O (v/v 7 : 3).

### 3.4. Optimization studies

To achieve optimal performance, several experimental parameters were systematically evaluated using l-Cys as a model analyte. The probe response was quantified in terms of the fluorescence enhancement, Δ*F* = *F* − *F*_0_, where *F* and *F*_0_ represent the fluorescence intensities in the presence and absence of l-Cys, respectively.

The effect of reaction time was first investigated. Fluorescence measurements were recorded at 5 min intervals up to 15 min (Fig. S8a). The maximum Δ*F* was observed at 10 min, after which the signal intensity plateaued. On this basis, we infer that a 10 min incubation would provide for a reliable operational timeframe. The influence of pH on the probe performance was also examined using the Britton–Robinson buffer over a pH range of 2–12 (Fig. S8b). A pronounced pH dependence was observed, with the maximum Δ*F* recorded at near-physiological pH (Britton–Robinson Buffer (BRB) pH 7). Under both strongly acidic and strongly basic conditions, the analyte-induced fluorescence enhancement decreased, likely due to protonation/deprotonation effects altering the probe–thiol reaction. These findings lead to the prediction that probe P3 is best suited for use under near-neutral conditions, as found in many biological environments.

### 3.5. Analytical performance of the P3 probe

#### 3.5.1 Fluorescence titrations with free thiols

Under optimized conditions (5% DMSO in pH 7 Britton–Robinson buffer, v/v- and 10-minutes incubation), a concentration-dependent increase in the fluorescence intensity was observed upon the incremental addition of l-Cys (Fig. 3a). To assess systematically the analytical performance of probe P3, standard l-Cys solutions were incubated with the probe solution for 10 min, and the fluorescence enhancement (Δ*F* = *F* − *F*_0_) was quantified. Per our design expectations, the reaction of l-Cys with P3 resulted in a readily discernible fluorescence enhancement. A linear calibration curve was generated within the concentration range of 10–60 µM (Fig. S9). The limit of detection (LOD), calculated based on the 3*σ*/slope method in accordance with analytical validation protocols,^35,36^ was found to be 5.32 µM.

**Fig. 3:**
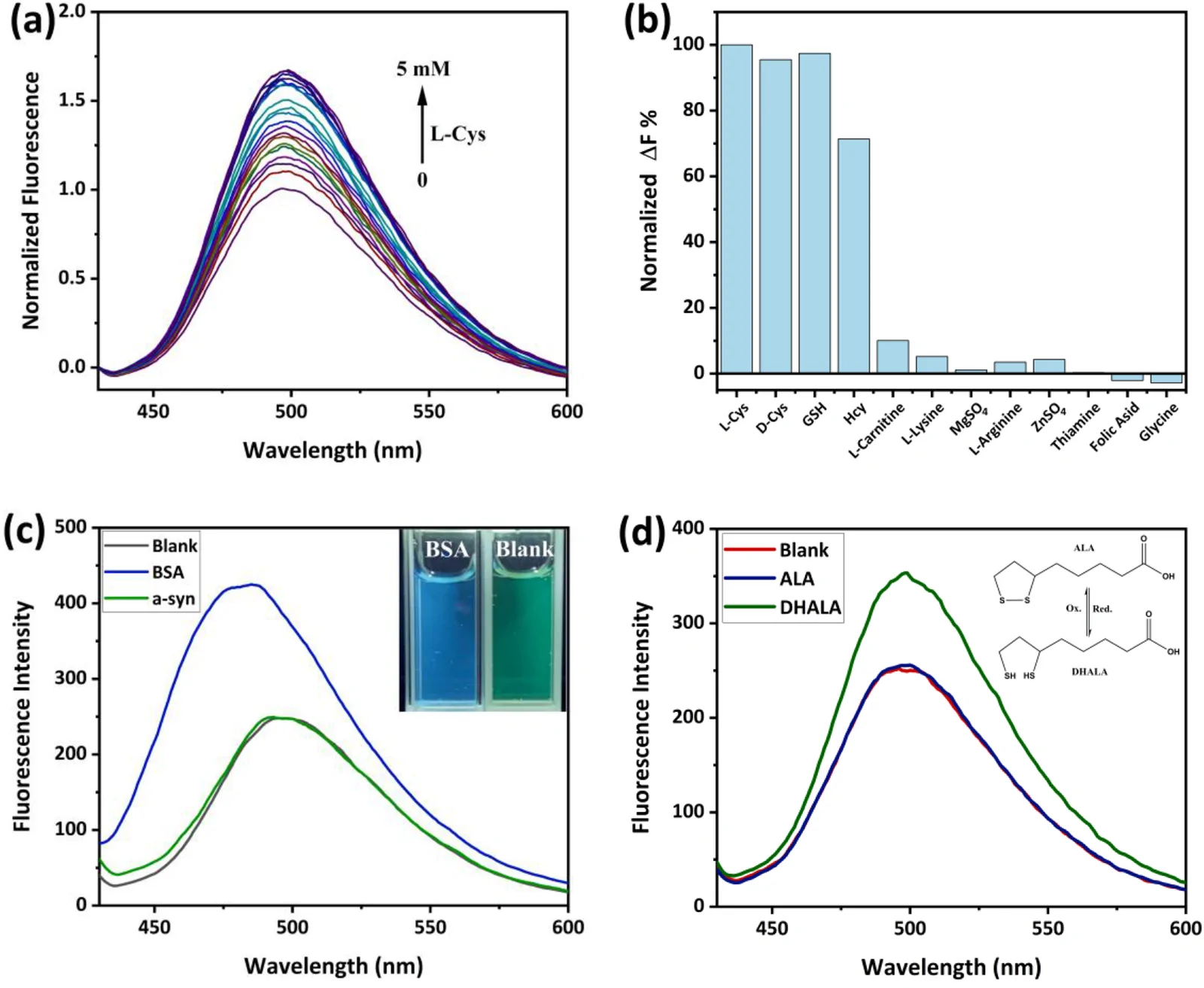
(a) Impact of varying l-Cys concentrations on the fluorescence intensity of 10 µM P3 at pH 7, (b) response of the probe to free thiols and other amino acids and compounds, (c) fluorescence changes upon interaction with α-synuclein and BSA, (d) fluorescence response to α-lipoic acid (ALA) before and after reduction to dihydrolipoic acid (DHALA) using sodium borohydride.

Complementary UV–Vis absorbance studies revealed a concentration-dependent decrease in the absorption intensity upon addition of l-Cys to P3 over the 0.1–7.5 mM concentration range (Fig. S10).

To evaluate the accuracy and precision of P3 as a probe for l-Cys, again used as a representative biothiol, intra- and inter-day recovery studies were performed. In this study, three different concentrations (30, 40, and 50 µM) of l-Cys solutions were prepared and tested in pH 7 Britton–Robinson buffer. Intra-day measurements were performed with three replicate studies being carried out on the same day, while inter-day measurements were conducted on the 1st, 3rd, and 5th days, again with three replicates each. The recovery ranged between 98% and 102% (Table S1), supporting the reliability, reproducibility, and robustness of the proposed probe system for routine l-Cys determinations.

Analogous studies were performed for GSH under optimized experimental conditions (5% DMSO in pH7 Britton–Robinson buffer, v/v- and 10-minutes incubation). As shown in Fig. S11a, adding increasing quantities of GSH to P3 induced a proportional increase in the fluorescence intensity. The calibration curve exhibited a linear response within the concentration range of 10–50 µM (Fig. S11b). The LOD for GSH was calculated as 5.94 µM, highlighting that P3 can detect effectively both l-Cys and GSH at low micromolar levels.

#### 3.5.2. Selectivity of the probe

A key requirement for a reliable probe is high selectivity toward the target analyte. Therefore, to evaluate the putative selectivity of P3, a range of possible substrates were tested under optimized conditions. For this purpose, different amino acids and compounds commonly found in plasma samples or dietary supplements including carnitine, lysine, arginine, folic acid, glycine, thiamine, ZnSO_4_, and MgSO_4_ were tested. The relative fluorescence response was recorded as normalized Δ*F* (%), where Δ*F* = *F* − *F*_0_. The maximum fluorescence change obtained from l-Cys (Δ*F*_MAX_) was set to 100%, and all values were normalized accordingly using the formula: normalized Δ*F*(%) = ((*F* − *F*_0_)/Δ*F*_MAX_) × 100%. Thiol-containing analytes showed comparable responses, while non-thiol-containing compounds engendered a <10% relative response (Fig. 3b). These findings provide further support for the suggestion that probe P3 could have a role to play in practical biothiol sensing applications.

#### 3.5.3. Detection of l-Cys in a food supplement

The use of l-Cys in dietary supplements is widespread, and its beneficial effects on human health have been well documented.^37^ In this study, we tested probe P3 in the analysis of a commercial dietary supplement (containing biotin, l-Cys, and zinc). To evaluate the accuracy and reliability of the tests based on P3, the results were compared with those obtained using the standard Ellman's reagent method. A statistical analysis (*t*-test) revealed a high level of agreement between the two approaches with a comparable level of accuracy being seen in both cases (Table S2). These results thus highlight further not only the robustness of P3 as a probe but also its potential for routine applications in the quality control of dietary supplements.

### 3.6. Detection of free thiols in small molecules and proteins

Free thiol (–SH) groups play a crucial role in maintaining the structural integrity and biological function of proteins. Because they are directly involved in the formation and reduction of disulfide bonds, the quantification of free thiols can serve as an indicator of a protein tertiary structure. Structural changes, such as unfolding or denaturation, can alter the accessibility and number of free thiol groups. Therefore, the sensitive and selective detection of thiols can provide potentially valuable insights into protein folding states and in favorable cases can facilitate tertiary structure characterization.^38,39^

As a first step to testing whether probe P3 could prove useful in this regard, α-synuclein (a model protein without any free thiol groups) was added to P3 (3 mL, 10 µM) in Britton–Robinson Buffer (pH 7) : DMSO (95 : 5). As shown in Fig. 3c, no significant change in fluorescence intensity was observed. In contrast, the addition of BSA, a free cysteine-containing protein, resulted in a pronounced increase in the fluorescence intensity. A hypsochromic shift in the emission spectrum was also observed with the fluorescence color changing from green to blue. While further study is warranted, we ascribe this shift to the encapsulation of P3 within the BSA pockets through non-covalent interactions, providing a more rigid microenvironment shielded from the solvent, followed by reaction with the adjacent free thiol group, Cys-34.

Probe P3 was also tested with ALA, a small molecule containing a disulfide bond. As expected, no significant fluorescence response was observed. However, the addition of sodium borohydride to this solution leads to cleavage of the disulfide bonds in ALA to produce dihydrolipoic acid (DHALA) and unmasking of free thiols. As expected, this reduction led to a marked increase in the fluorescence intensity of probe P3, as shown in Fig. 3d.

Taken in concert, the above results provide support for the design expectation that probe P3 can selectively detect free thiols in both proteins and small molecules. The nature of the response leads us to suggest that it could see application in probing specific microenvironments, as well as potentially tertiary structural differences and conformational changes in proteins.

### 3.7. Applications in living cell imaging

The sensitivity of P3 probe to free thiol levels led us to explore its use in distinguishing between cancerous and healthy cells. Cancer cells typically display elevated concentrations of thiol-containing biomolecules, such as glutathione (*vide supra*). Probe P3 was thus expected to provide a selective response to cancerous *vs.* normal cell types. To the extent this proved true, it would thus make probe P3 an effective tool for cellular imaging. An effort was thus made to test this possibility.

To minimize cytotoxic effects and ensure high cell viability throughout the experiments, probe P3 was applied to cells using low levels of an organic solvent (DMSO). The probe concentration and objective of the microscope were optimized for the discrimination of cancer and healthy cells (Fig. S12). Upon incubation with P3, a distinct fluorescence signal was observed in the case of cancer cells, whereas healthy cells under identical conditions exhibited only negligible fluorescence (*cf.*Fig. 4). In fact, the contrast between cancerous and normal cell lines is readily apparent upon casual observation. For instance, colon cancer cells (HT29, Fig. 4a) displayed a strong green fluorescence, while the healthy colon epithelial cells (FHC, Fig. 4b) remained almost non-fluorescent. Similarly, in lung cancer cells (A549, Fig. 4c), a pronounced fluorescence signal was observed; in contrast, little signal was seen for healthy lung epithelial cells (BEAS-2B, Fig. 4d). This capability to differentiate between malignant and healthy proved reproducible across multiple concentrations and magnifications, with optimal conditions determined for each cell line (*e.g.*, 5 µM P3 at 20× magnification for A549 cells, and 2.5 µM P3 at 20× magnification for HT29 cells). Overall, the performance of P3 in live-cell imaging highlights its potential not only as an analytical probe for biothiol detection but also as a potentially useful diagnostic tool for visualizing cancer cells.

**Fig. 4:**
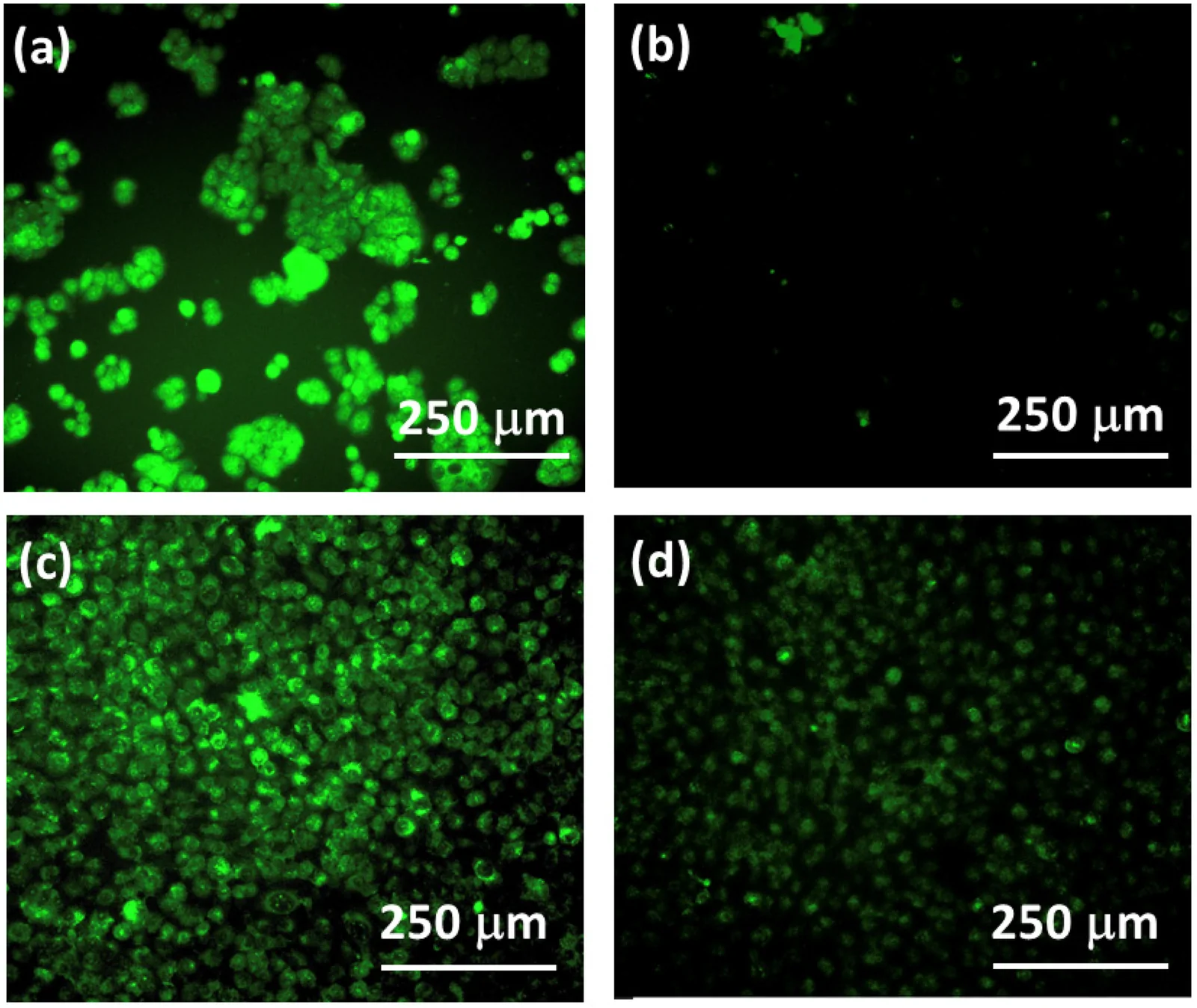
Fluorescence microscopy images of cells at 20× magnification after different treatments. (a) Cancerous colon cells (HT-29) with 5 µM P3. (b) Healthy colon cells (FHC) with 5 µM P3, (c) cancerous lung cells (A549) with 2.5 µM P3, (d) healthy lung cells (BEAS-2B) with 2.5 µM P3.

To examine the intracellular distribution of P3 at higher spatial resolution, HT-29 cells were imaged using confocal microscopy (Fig. S13). This revealed an intracellular signal that was punctate and granular. This was taken as evidence that P3 preferentially accumulates within discrete cytoplasmic regions, potentially in specific intracellular organelles.

### 3.8. Perceived advantages of P3 as a thiol probe

To date, a range of detection mechanisms have been exploited in the development of thiol probes. These include aromatic substitution reactions, rearrangements, aldehyde cyclizations, Michael additions, Se–N or S–S bond-forming or cleavage reactions, cyclizations, reactions with acrylates, interactions with metal complexes, and nucleophilic substitution reactions.^24^ The present coumarin-quinolinium hybrid fluorescent probe (P3) distinguishes itself from these prior efforts in that it exploits a unique merocyanine-spirocyclic transformation as its sensing mechanism. As a result, P3 is highly sensitive to environmental conditions, including pH and solvent polarity. In operational terms, the inherent responsiveness of P3 translates into a strong turn-on fluorescent enhancement in the presence of biothiols.

While several existing thiol probes offer low limits of detection (LODs), many suffer from poor aqueous compatibility and a lack of broad applicability (Table S3). One advantage of P3 over these previously reported fluorescent probes is its aqueous compatibility. It operates effectively in a low-organic-solvent medium (DMSO/BRT buffer = 1 : 20 v/v, pH 7.0). This contrasts with many thiol-sensing probes that necessitate higher organic solvent content (*e.g.*, 20–50% ACN or EtOH). The ability of P3 to function optimally under near-physiological conditions allows its use in biological applications. Additionally, the probe exhibits a considerable Stokes shift of 90 nm. This shift helps minimize spectral overlap between the excitation and emission, thereby reducing background interference and enhancing the signal-to-noise ratio.

Another feature of P3 is its versatility across a range of applications. It shows promise in food supplement analysis and in characterizing protein tertiary structures. It also allows effective differentiation between cancerous (HT-29, A549) and healthy (FHC, BEAS-2B) cell lines. The reversible nature of its spirocyclization mechanism, where water facilitates the ring-opening to regenerate the original P3 structure, offers the potential for dynamic monitoring of thiol fluctuations in complex biological systems. We thus suggest that probe P3 could emerge as a useful tool for those seeking to monitor biothiol concentrations and the attendant physiological consequences.

## 4. Conclusions

In this study, a coumarin-based probe (P3) was synthesized and found to be an efficient optical probe for biothiols as confirmed by spectrophotometric analyses. The synthetic intermediate P2 (a salicylaldehyde derivative bearing a coumarin substituent at the 5^th^ position) was obtained in one step from 4-(7-(diethylamino)-2-oxo-2*H*-chromen-3-yl) phenyl acetate (compound P1). Conversion to P3 then proved facile.

Probe P3 produced a strong fluorescence response when exposed to free thiol-containing molecules, such as cysteine and glutathione. Test involving various amino acids, vitamins, and constituents of plasma revealed specificity for thiol-containing substrates, as well as good stability and reliability. The observed response to thiol levels allowed insights into the tertiary structure and conformational stability of proteins to be inferred. *In vitro* studies further revealed that probe P3 could be used to differentiate between cancerous and healthy cells, a finding ascribed to differences in free biothiol levels.

Taken together, these features position P3 as a promising and practical fluorescent probe for use in biological contexts where determination of free thiol-containing biomarker levels can translate into useful analytical and diagnostic understanding.

## Author contributions

Jonathan L. Sessler: writing – review & editing, supervision, formal analysis. Mohammed Adnan Abdulsalam Abdulsalam: writing – review & editing, visualization, investigation, formal analysis, data curation, conceptualization. Halil Duyar: writing – original draft, visualization, validation, software, investigation, data curation, conceptualization. Ferda Hacivelioglu: writing – review & editing, writing – original draft, visualization, formal analysis, data curation, conceptualization. Burak Derkus: writing – review & editing, visualization, investigation. Ergin Yalcin: writing – original draft, visualization, validation, software, investigation, data curation, conceptualization, experimentation. Mehmet Gokhan Caglayan: writing – review & editing, writing – original draft, visualization, supervision, project administration, methodology, investigation, funding acquisition, formal analysis, data curation, conceptualization.

## Conflicts of interest

The authors declare that they have no known competing financial interests or personal relationships that could appear to influence the work reported in this paper.

## Supplementary Material

AN-OLF-D6AN00818F-s001

## Data Availability

Supplementary information (SI): NMR spectra, mass spectra, fluorescence spectroscopic data, details of method validation and optimization experiments, statistical tests and results of the cell imaging studies. See DOI: https://doi.org/10.1039/d6an00818f.

## References

[cit1] Lai Y. T., Ganguly A., Chen L. C., Chen K. H. (2010). Biosens. Bioelectron..

[cit2] Zhang X., Li G., Pan W., Li N., Li Y., Tang B. (2024). Chem. Res. Chin. Univ..

[cit3] Ghobadloo P. A. , Keles G., Yarman A., Hamidi S. and Kurbanoglu S., in Surfactant Based Electrochemical Sensors and Biosensors, Elsevier, 2024, pp. 315–342

[cit4] Riemer J., Schwarzländer M., Conrad M., Herrmann J. M. (2015). Biol. Chem..

[cit5] Ventura S. and Chang R. J. Y., in Folding of Disulfide Proteins, ed. R. J. Y. Chang and S. Ventura, Springer, New York, 2011, pp. 1–22

[cit6] Semenovich D. S., Plotnikov E. Y., Titko O. V., Lukiyenko E. P., Kanunnikova N. P. (2021). Antioxidants.

[cit7] Akunal C., Alisik M. (2024). Clin. Biochem..

[cit8] Kuiper J. M., Pluta R., Huibers W. H. C., Fusetti F., Geertsma E. R., Poolman B. (2009). Protein Sci..

[cit9] Koo B. K., Munroe W., Gralla E. B., Valentine J. S., Whitelegge J. P. (2021). Front. Neurosci..

[cit10] Mondal A., Pareek N., Khan A. R., Sahoo S. K., Samanta A., Dutta S. (2025). Analyst.

[cit11] Mahrose N. E., Hassan F. A., Jebali J., Mohammed S. (2024). Al-Nahrain J. Sci..

[cit12] Huang S., Yin H. (2025). Biomedicines.

[cit13] Chen W., Zhao Y., Seefeldt T., Guan X. (2008). J. Pharm. Biomed. Anal..

[cit14] Ling Y. Y., Yin X. F., Fang Z. L. (2005). Electrophoresis.

[cit15] Calvo-Marzal P., Chumbimuni-Torres K. Y., Höehr N. F., Kubota L. T. (2006). Clin. Chim. Acta.

[cit16] Nezamzadeh-Ejhieh A., Hashemi H. S. (2012). Talanta.

[cit17] Wang P., Wang Q., Huang J., Li N., Gu Y. (2017). Biosens. Bioelectron..

[cit18] Waseem A., Yaqoob M., Nabi A. (2008). Luminescence.

[cit19] Xu K., Zhang Y., Tang B., Laskin J., Roach P. J., Chen H. (2010). Anal. Chem..

[cit20] Xing J., Zhou G., Sun C., Zhang H., Chen B., Zong X., Cai J., Ji M. (2016). Biotechnol. Lett..

[cit21] Liang Z., Jiang Z., Zhang C., Liu Z. (2023). J. Mol. Struct..

[cit22] Zhang H., Wang C., Wang K., Xuan X., Lv Q., Jiang K. (2016). Biosens. Bioelectron..

[cit23] Wang H., Zhang P., Zhang C., Chen S., Zeng R., Cui J., Chen J. (2020). Mater. Adv..

[cit24] Asghar R., Li Y., Huo F., Yin C. (2024). Chem. Biomed. Imaging.

[cit25] Zhang R., Yong J., Yuan J., Xu Z. P. (2020). Coord. Chem. Rev..

[cit26] de la Fuente Revenga M., Herrera-Arozamena C., Fernández-Sáez N., Barco G., García-Orue I., Sugden D., Rivara S., Rodríguez-Franco M. I. (2015). Eur. J. Med. Chem..

[cit27] Wu W. N., Wu H., Wang Y., Mao X. J., Zhao X. L., Xu Z. Q., Fan Y. C., Xu Z. H. (2018). Spectrochim. Acta, Part A.

[cit28] Hu Y. H., Yang J., Zhang Y., Liu K. C., Liu T., Sun J., Wang X. J. (2019). J. Enzyme Inhib. Med. Chem..

[cit29] Rauhamäki S., Postila P. A., Niinivehmas S., Kortet S., Schildt E., Pasanen M., Manivannan E., Ahinko M., Koskimies P., Nyberg N., Huuskonen P., Multamäki E., Pasanen M., Juvonen R. O., Raunio H., Huuskonen J., Pentikäinen O. T. (2018). Front. Chem..

[cit30] Wu L., Gardiner J. E., Kumawat L. K., Han H.-H., Guo R., Li X., He X.-P., Elmes R. B. P., Sedgwick A. C., Bull S. D., James T. D. (2019). RSC Adv..

[cit31] Ramamurthy K., Ponnusamy K., Chellappan S. (2020). RSC Adv..

[cit32] Mandal M., Banik D., Karak A., Manna S. K., Mahapatra A. K. (2022). ACS Omega.

[cit33] Kortekaas L., Chen J., Jacquemin D., Browne W. R. (2018). J. Phys. Chem. B.

[cit34] Seiler V. K., Callebaut K., Robeyns K., Tumanov N., Wouters J., Champagne B., Leyssens T. (2018). CrystEngComm.

[cit35] ICH Harmonised Tripartite Guideline , Validation of Analytical Procedures: Text and Methodology Q2(R1), *International Conference on Harmonisation*, 2014

[cit36] Ozkan S. A. , Kauffmann J.-M. and Zuman P., Electroanalysis in Biomedical and Pharmaceutical Sciences, Springer, Berlin, Heidelberg, 2015, ch. 8, pp. 235–266

[cit37] Pucciarini L., Saluti G., Galarini R., Carotti A., Macchiarulo A., Rudaz S., Sardella R. (2020). J. Pharm. Biomed. Anal..

[cit38] Putzu M., Gräter F., Elstner M., Kubař T. (2018). Phys. Chem. Chem. Phys..

[cit39] Maag D., Putzu M., Gómez-Flores C. L., Gräter F., Elstner M., Kubař T. (2021). Phys. Chem. Chem. Phys..

