# Supplementary material for: Coumarin-quinolinium based fluorescent probe for free thiol detection and cancer cell imaging
Source: Analyst. 2026 Nov 5. Online ahead of print. doi: 10.1039/d6an00818f (PMC13761697; doi:10.1039/d6an00818f)
Supplement: Supplementary file 1 [file AN-OLF-D6AN00818F-s001.pdf]

Supplementary Information (SI) for Analyst.
This journal is © The Royal Society of Chemistry

Supporting Information for:

Coumarin-Quinolinium Based Fluorescent Probe for Free Thiol Detection and Cancer Cell Imaging

Mohammed Adnan Abdulsalam Abdulsalam,[ab] Halil Duyar,[c] Ferda Hacivelioglu[c], Burak Derkus[d], Jonathan L. Sessler,*[e] Ergin Yalcin*[ef] and Mehmet Gokhan Caglayan*[a]

[a.] *Ankara University, Faculty of Pharmacy, Department of Analytical Chemistry, Ankara, TURKIYE.*
[b.] *Ankara University, Graduate School of Health Sciences, TURKIYE.*
[c.] *Department of Chemistry, Gebze Technical University, Gebze, Kocaeli/TURKIYE.*
[d.] *Ankara University, Department of Chemistry, Ankara, TURKIYE*
[e.] *Department of Chemistry, The University of Texas at Austin, 105 East 24th Street-A5300, Austin, Texas 78712-1224, United States.*
[f.] *Department of Engineering Basic Sciences, Faculty of Natural Sciences and Engineering, Iskenderun Technical University, Hatay/ TURKIYE.*

Structural and spectroscopic characterization of P1 2

Structural and spectroscopic characterization of P2 3

Structural and spectroscopic characterization of P3 5

Solvatochromism of P3 7

NMR titration of P3 and L-Cys 7

Optimization of incubation time and pH for fluorescence enhancement 8

Calibration curve 10

UV-Vis titration of P3 and L-Cys 10

Fluorescence titration of P3 and Glutathione 11

Analytical method validation results 11

Optimization of P3 concentration and microscope objective magnification for fluorescence cell imaging 12

Confocal fluorescence images of HT-29 cells stained with P3 13

Comparison table for thiol detection 14

References 15

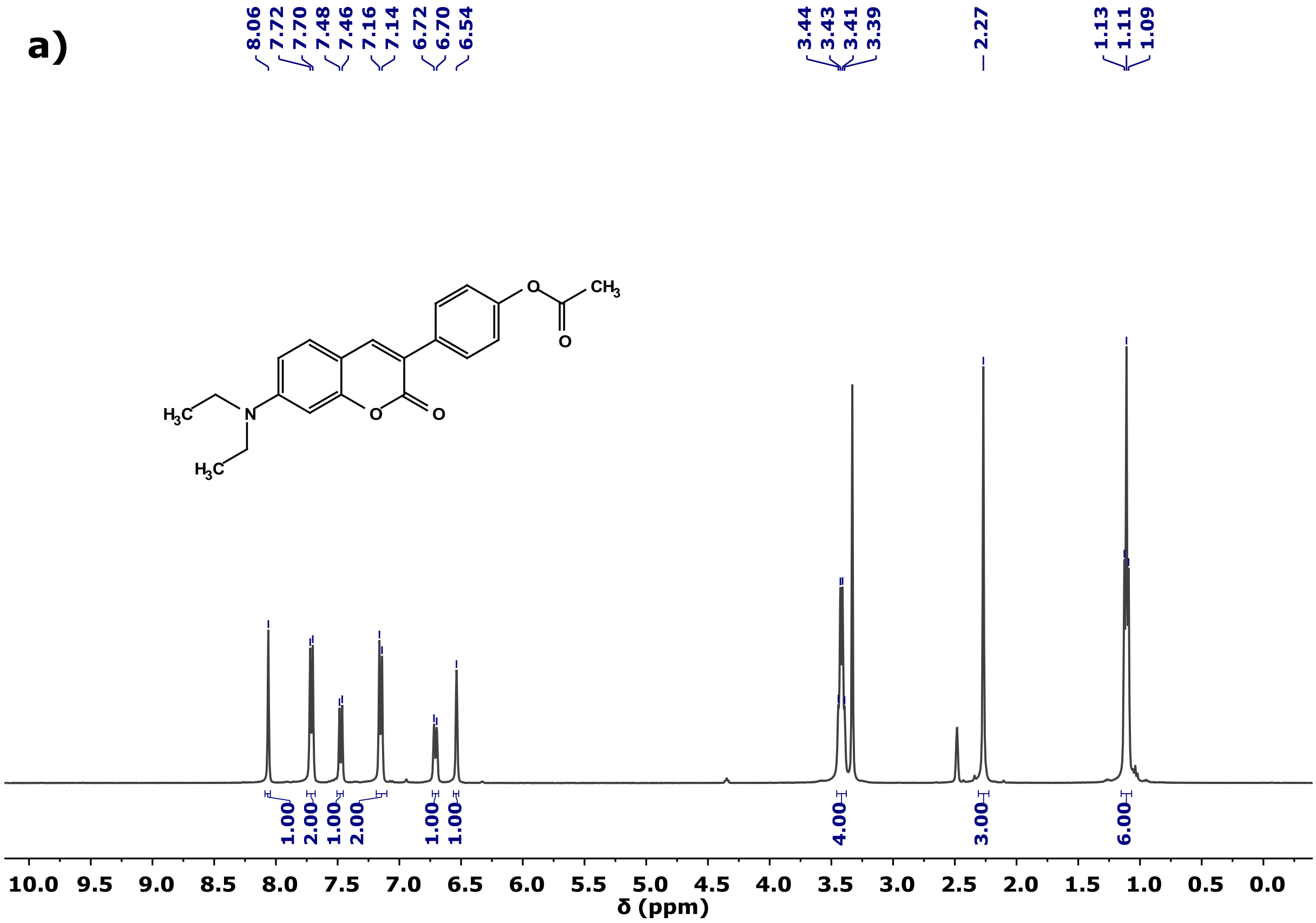

Fig. S1. a) ^{1}H NMR spectrum (400 MHz, DMSO-d_6 298 K) of **P1**

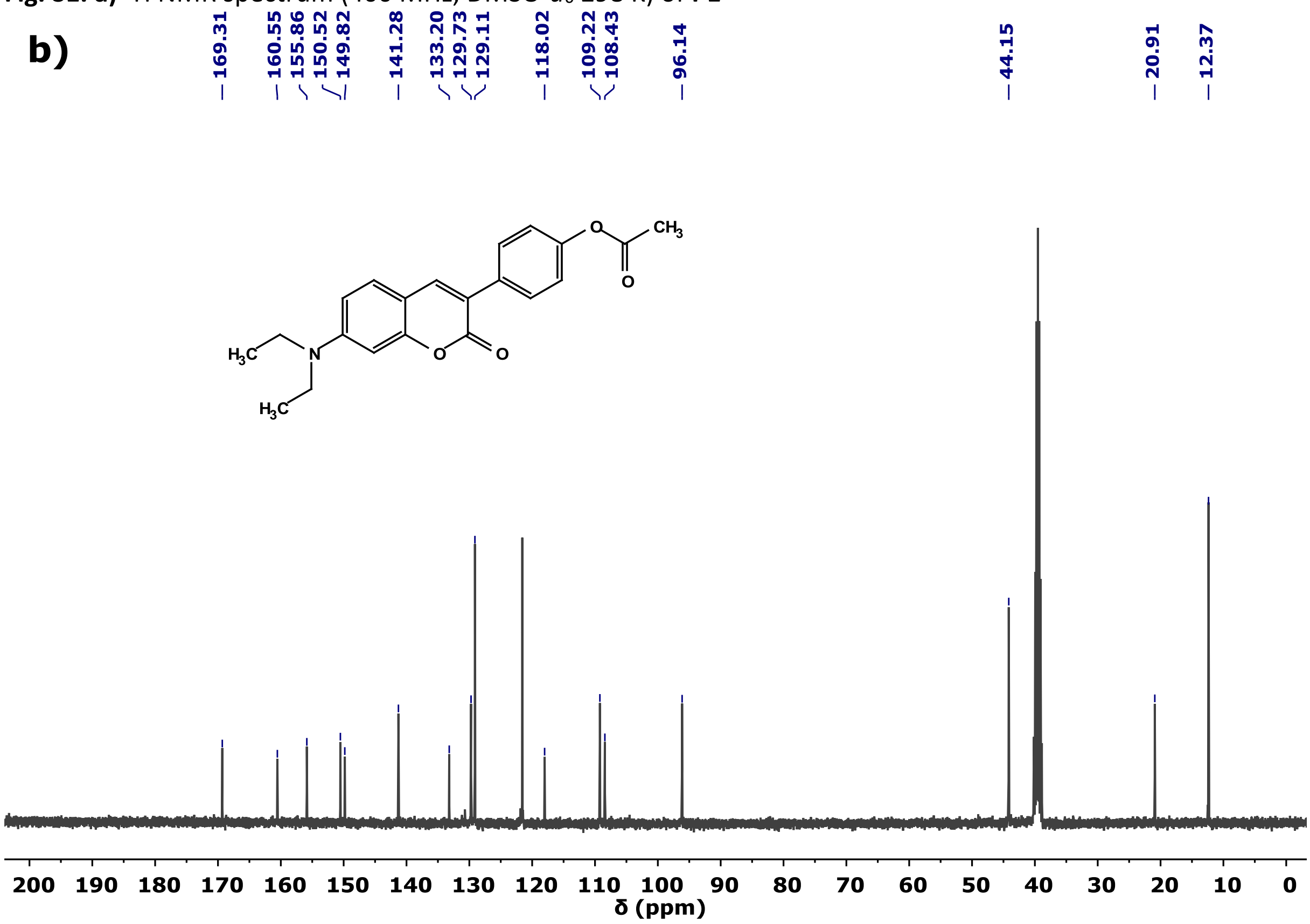

Fig. S1. b) ^{13}C NMR spectrum (100 MHz, DMSO-d_6 298 K) of **P1**

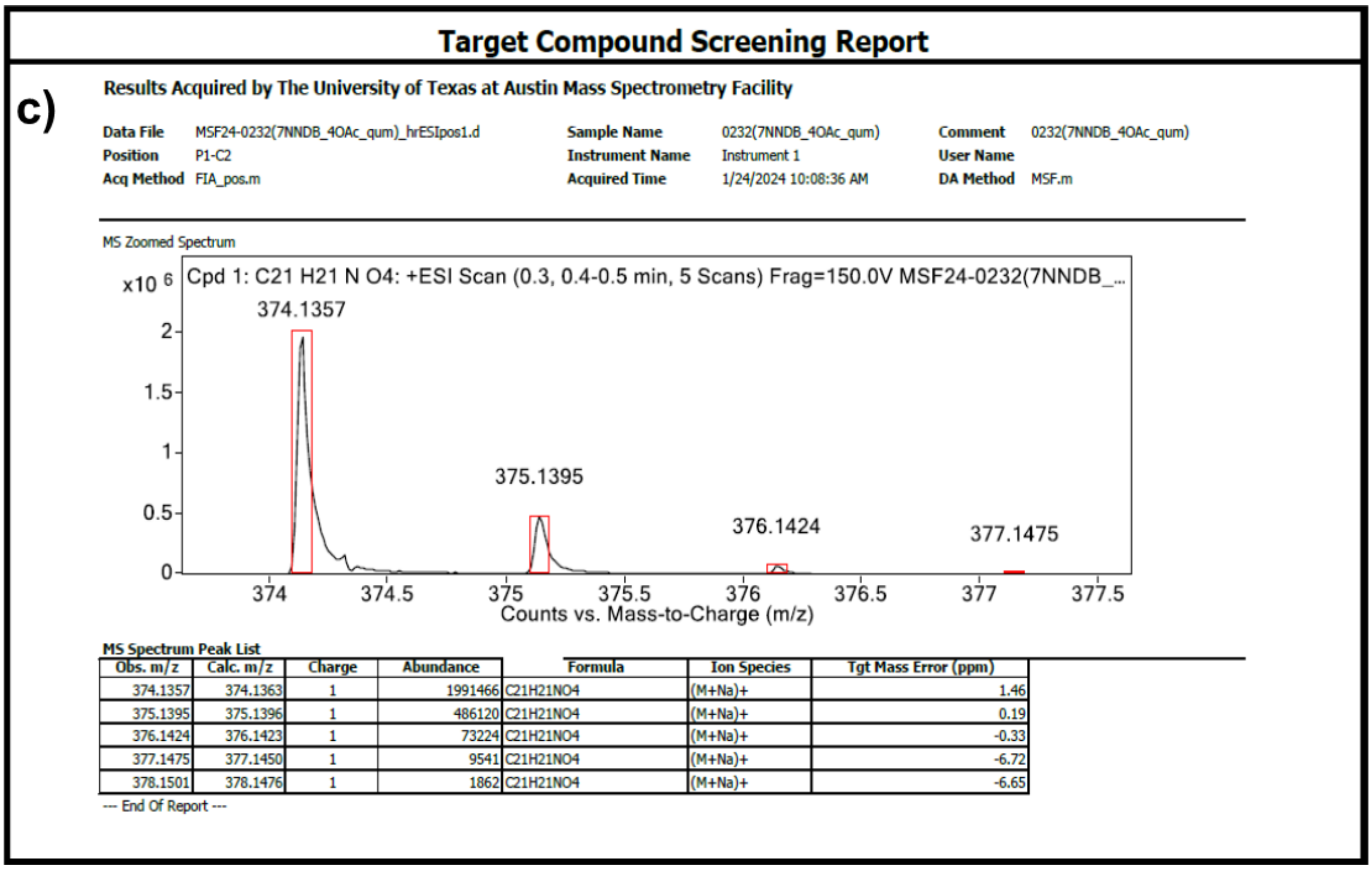

Obs. m/z	Calc. m/z	Charge	Abundance	Formula	Ion Species	Tgt Mass Error (ppm)
374.1357	374.1363	1	1991466	C21H21NO4	(M+Na)+	1.46
375.1395	375.1396	1	486120	C21H21NO4	(M+Na)+	0.19
376.1424	376.1423	1	73224	C21H21NO4	(M+Na)+	-0.33
377.1475	377.1450	1	9541	C21H21NO4	(M+Na)+	-6.72
378.1501	378.1476	1	1862	C21H21NO4	(M+Na)+	-6.65

Fig. S1. c) HRMS spectrum of P1.

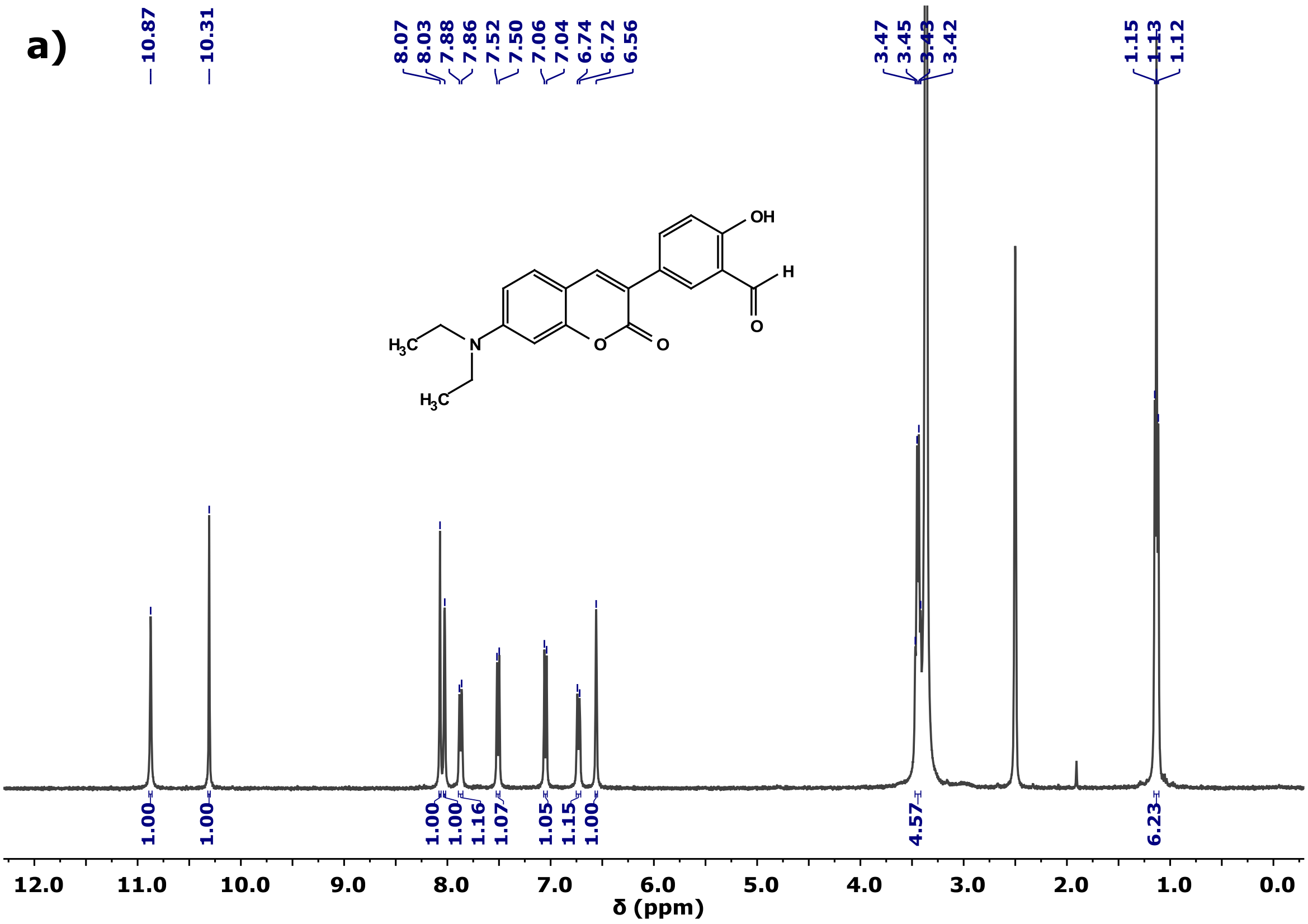

Fig. S2. a) ^{1}H NMR spectrum (400 MHz, DMSO-d_6 298 K) of **P2**

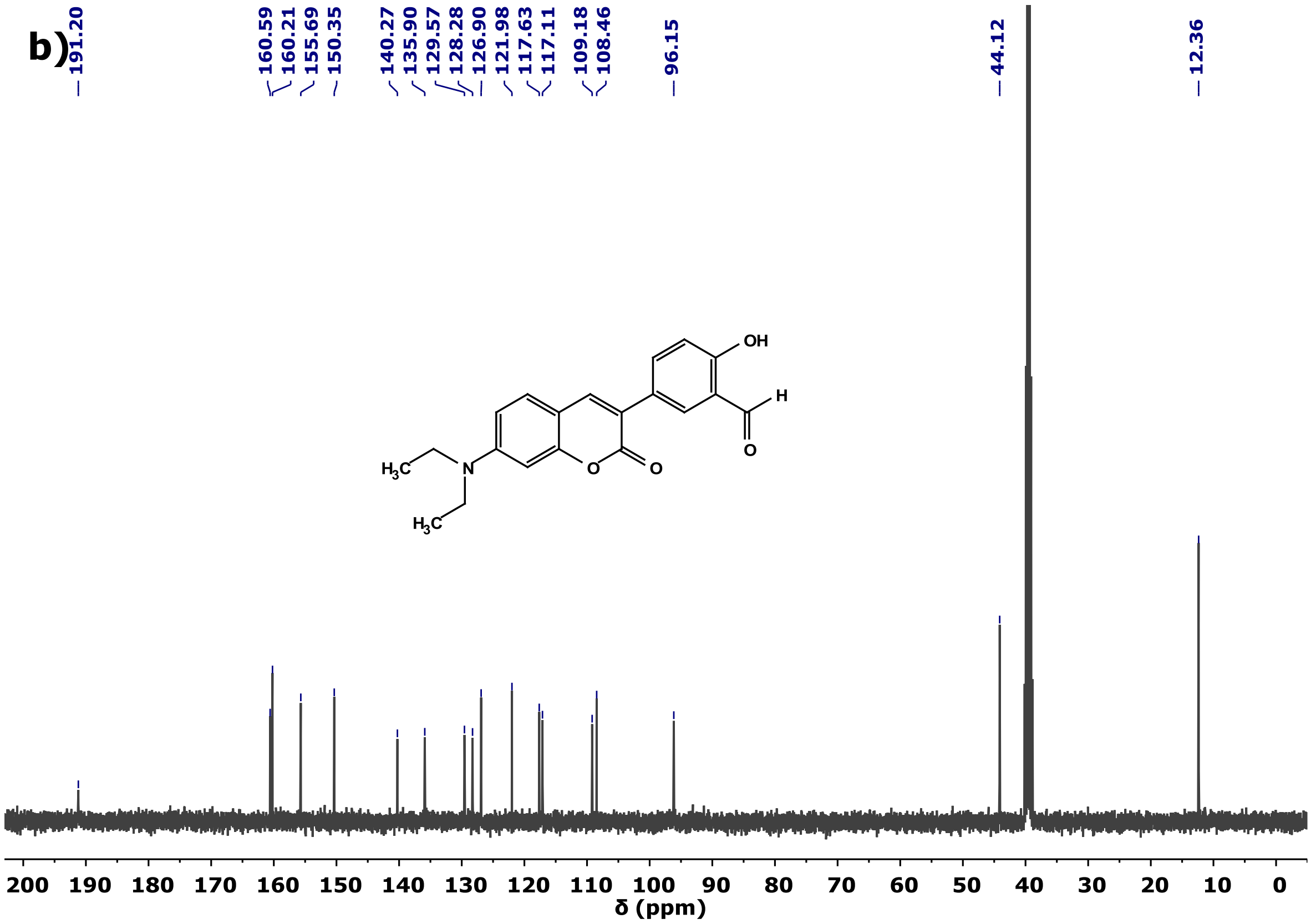

Fig. S2. b) ^{13}C NMR spectrum (100 MHz, DMSO-d_6 298 K) of **P2**

Target Compound Screening Report

c)

Results Acquired by The University of Texas at Austin Mass Spectrometry Facility

**Data File**	MSF24-0233(7NNDB_4OH_qald)_hrESIpos1.d	**Sample Name**	0233(7NNDB_4OH_qald)	**Comment**	0233(7NNDB_4OH_qald)
**Position**	P1-C3	**Instrument Name**	Instrument 1	**User Name**	
**Acq Method**	FIA_pos.m	**Acquired Time**	1/24/2024 10:10:39 AM	**DA Method**	MSF.m

MS Zoomed Spectrum

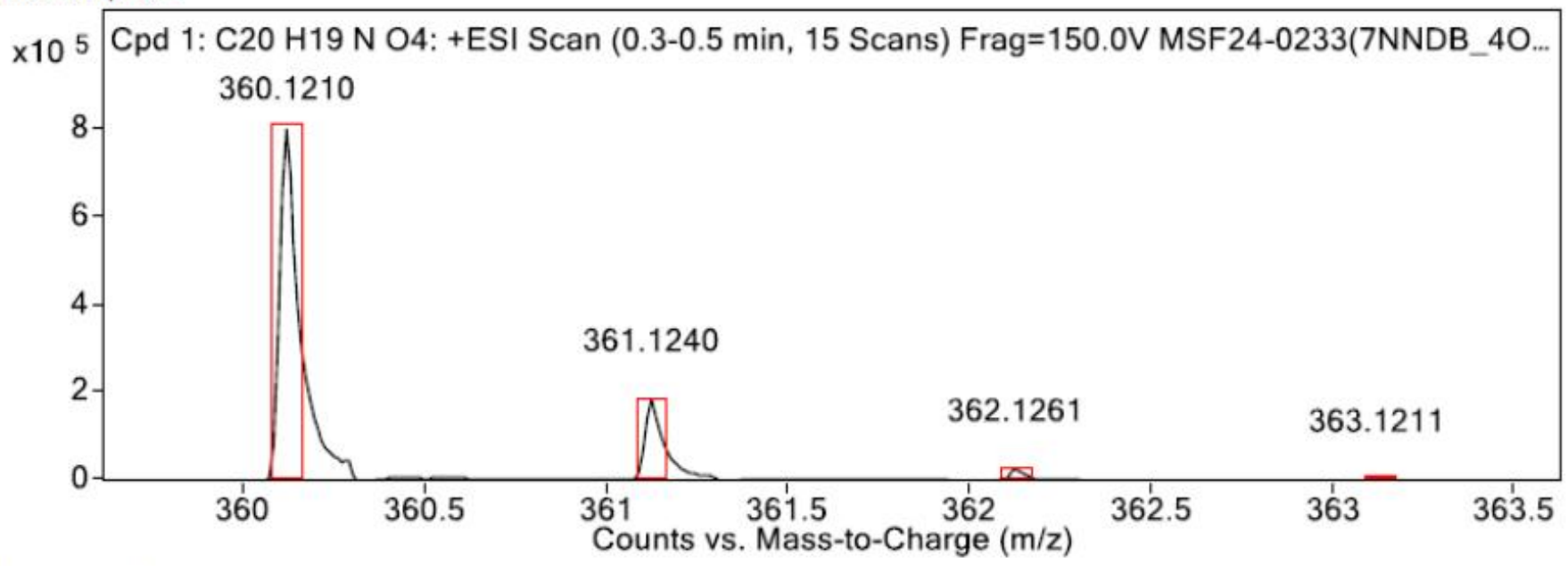

MS Spectrum Peak List

Obs. m/z	Calc. m/z	Charge	Abundance	Formula	Ion Species	Tgt Mass Error (ppm)
360.1210	360.1206	1	803215	C20H19NO4	(M+Na)+	-1.09
361.1240	361.1239	1	187573	C20H19NO4	(M+Na)+	-0.27
362.1261	362.1266	1	27525	C20H19NO4	(M+Na)+	1.47
363.1211	363.1292	1	3440	C20H19NO4	(M+Na)+	22.3
364.1067	364.1318	1	721	C20H19NO4	(M+Na)+	68.97

--- End Of Report ---

Fig. S2. c) HRMS spectrum of **P2**

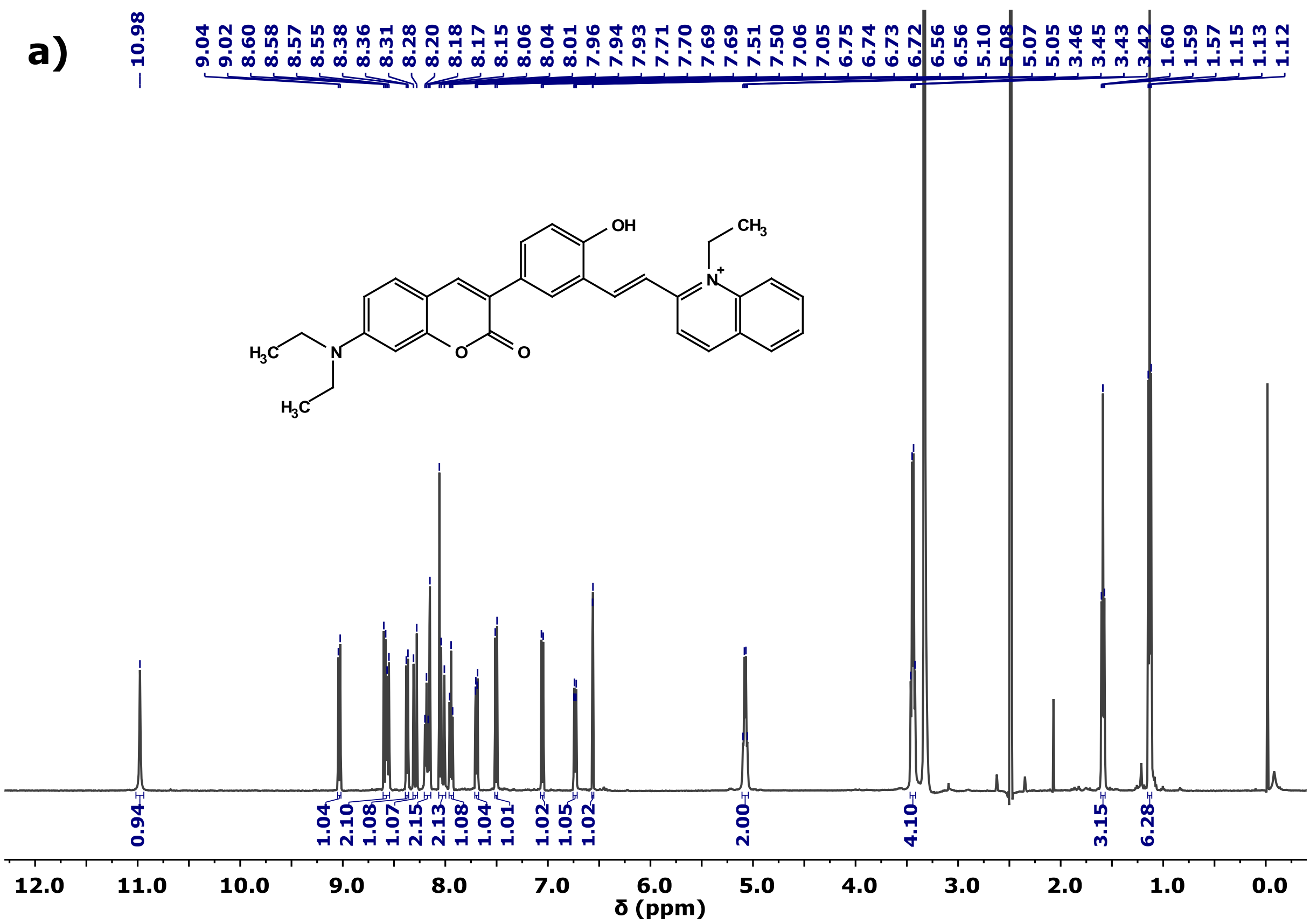

Fig. S3. a) ^{1}H NMR spectrum (500 MHz, DMSO-d_6 298 K) of **P3.**

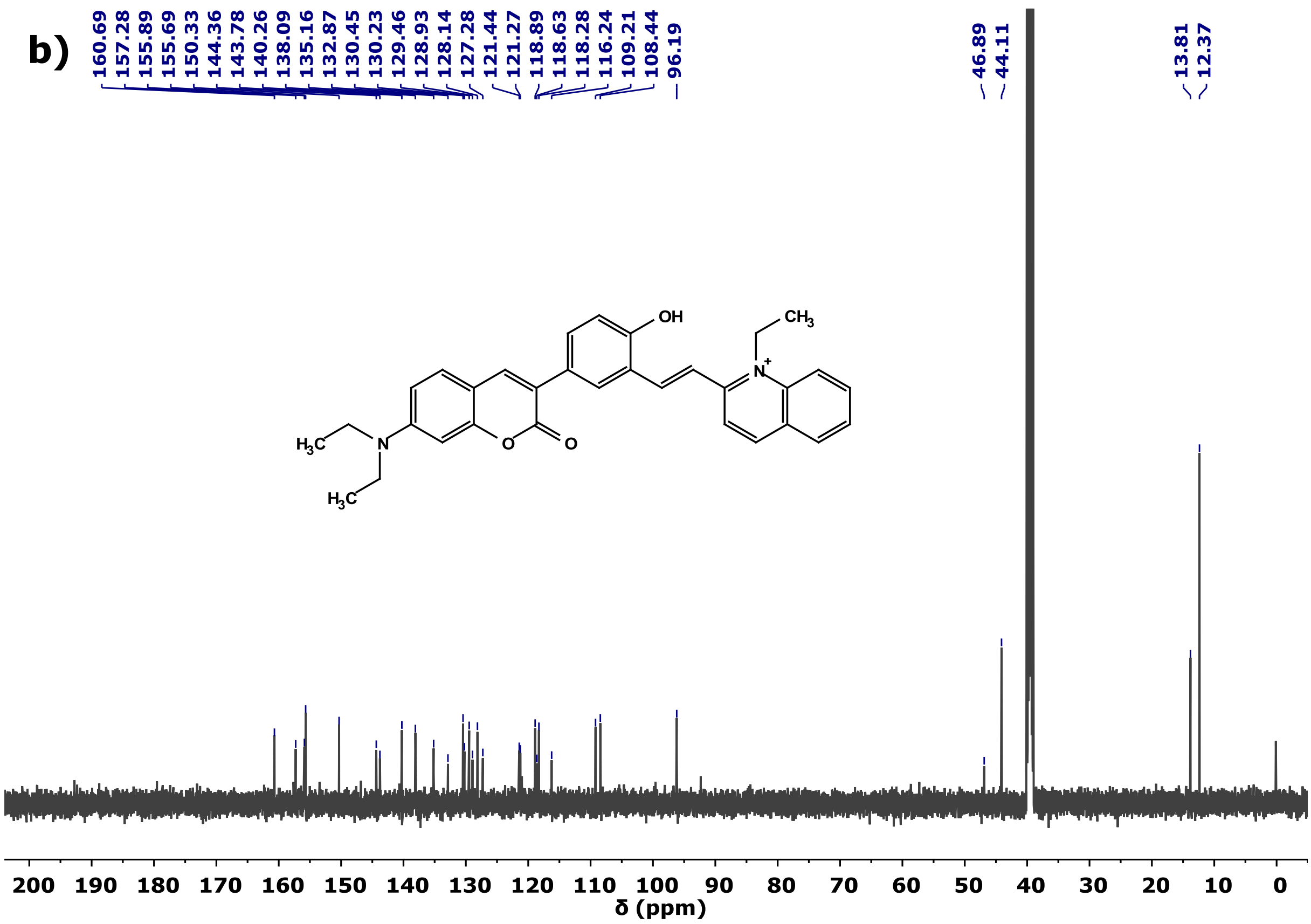

Fig. S3. b) ^{13}C NMR spectrum (125 MHz, DMSO-d_6 298 K) of **P3.**

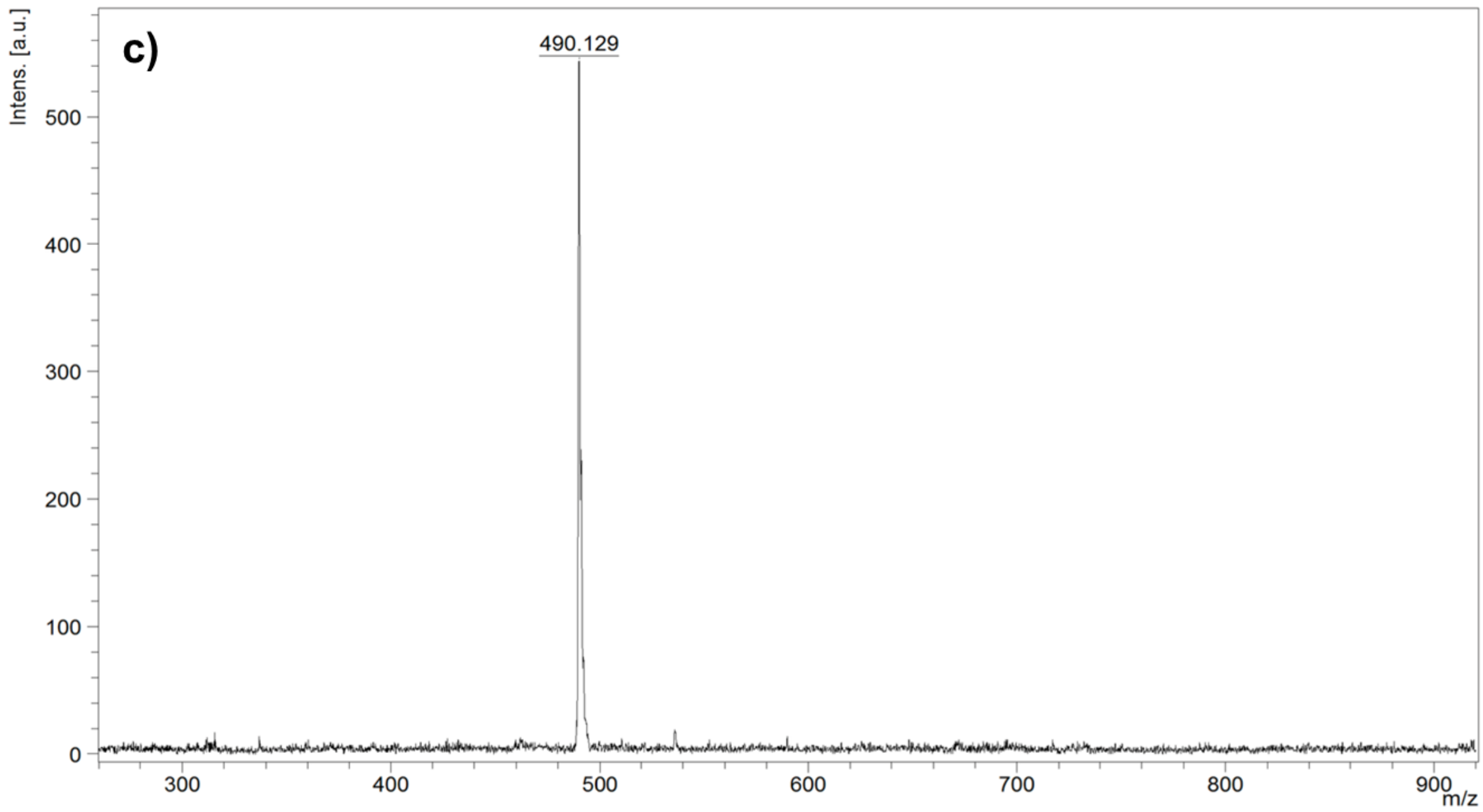

Fig. S3. c) HRMS spectrum of **P3**.

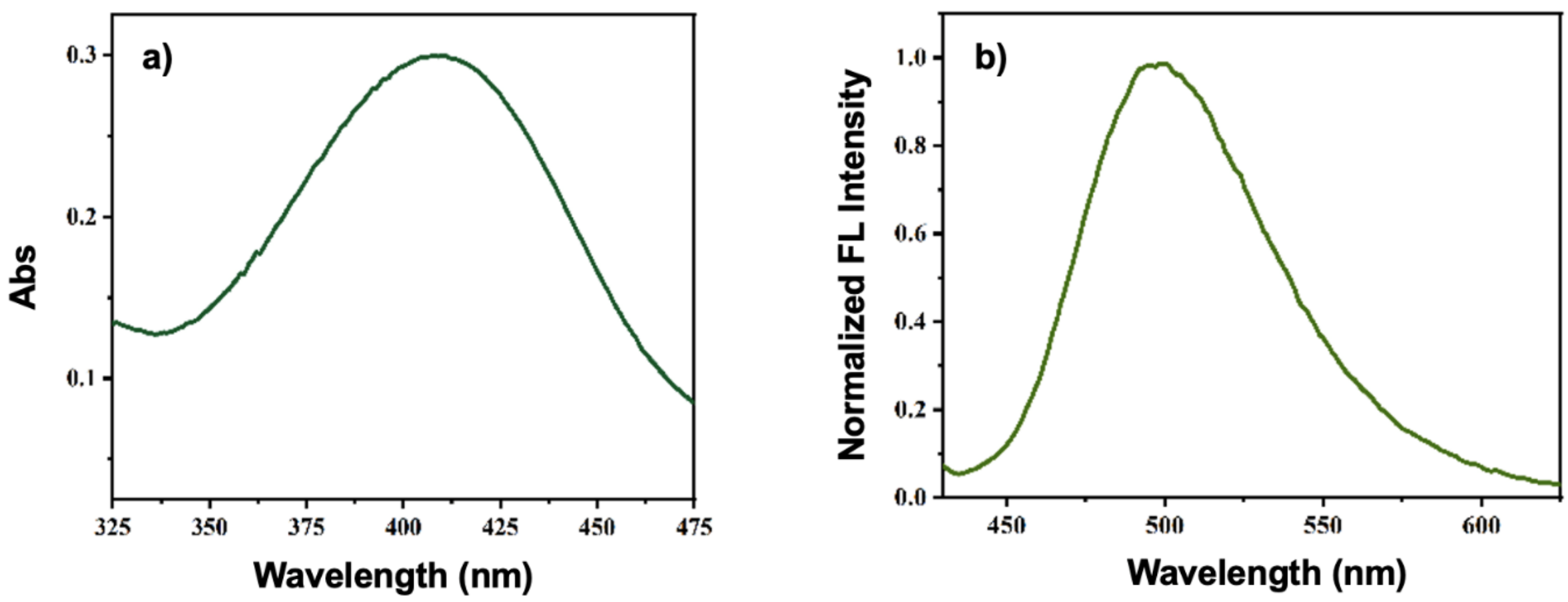

Fig. S4. Spectroscopic characterization of a 10 µM solution of **P3** in Britton-Robinson Buffer (pH 7): DMSO (95:5) **a)** UV-Vis absorbance spectrum and **b)** fluorescence spectrum

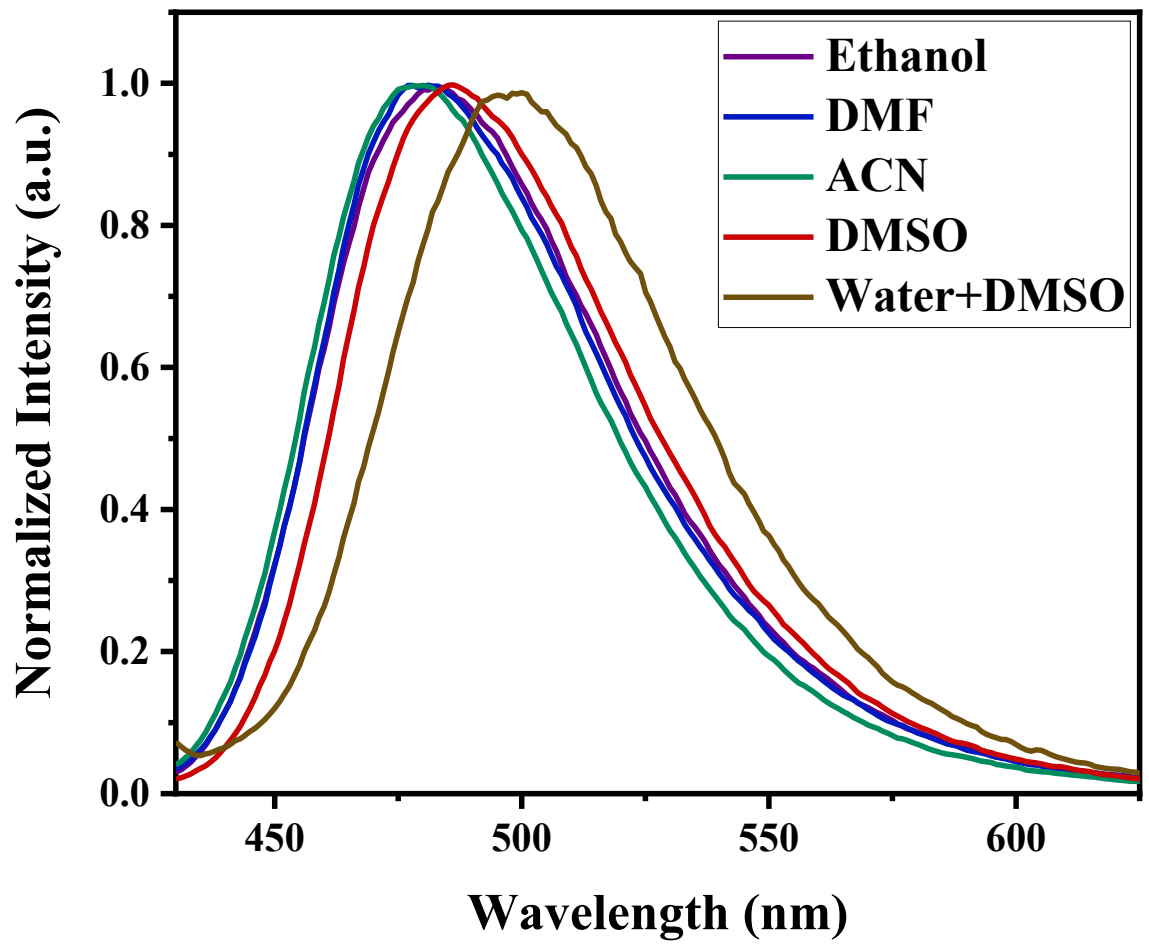

Fig. S5. Emission spectra of 10 µM **P3** recorded in the indicated solvents.

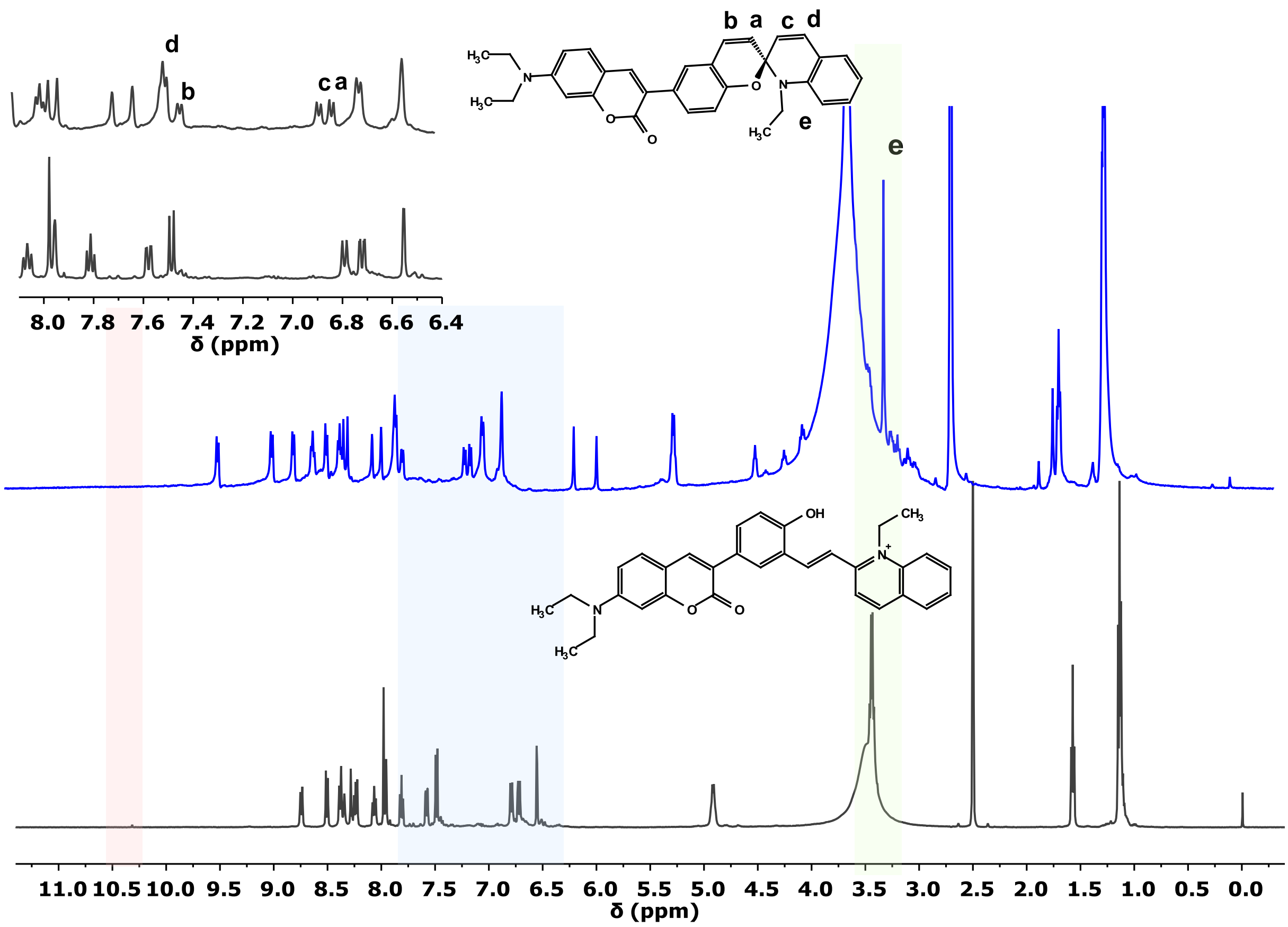

Fig S6. ^{1}H-NMR spectra of **P3** recorded in the absence and presence of L-Cys (3 equivalents) in DMSO-d_6

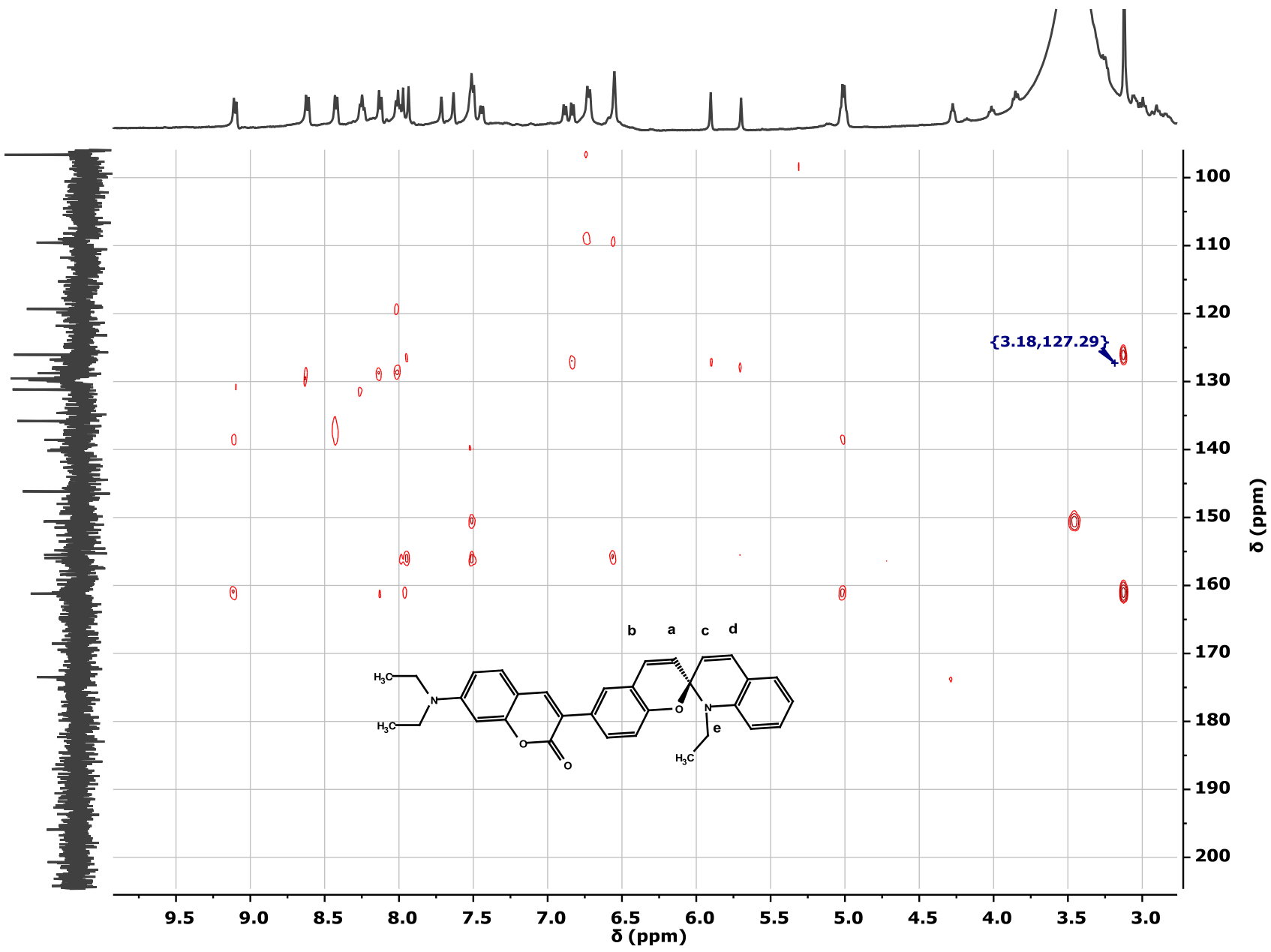

Fig S7. HMBC spectrum of **P3** recorded in presence of L-Cys (3 equivalents) in DMSO-d_6

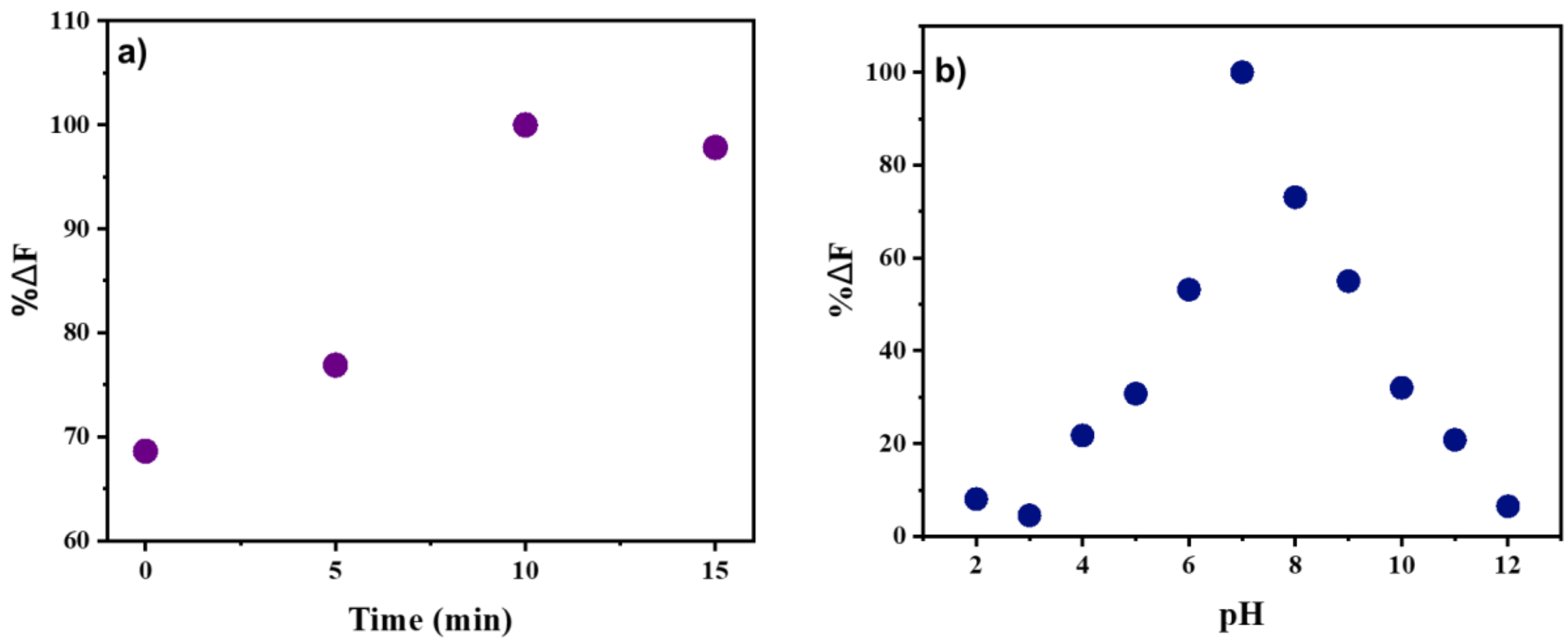

Fig. S8. Optimization of incubation time and pH for fluorescence enhancement. (a) Effect of incubation time of P3 and L-Cys on the normalized fluorescence enhancement. (b) Effect of pH on the normalized fluorescence enhancement. The fluorescence enhancement values were normalized by assigning the highest fluorescence enhancement value obtained in each experiment as 100%.

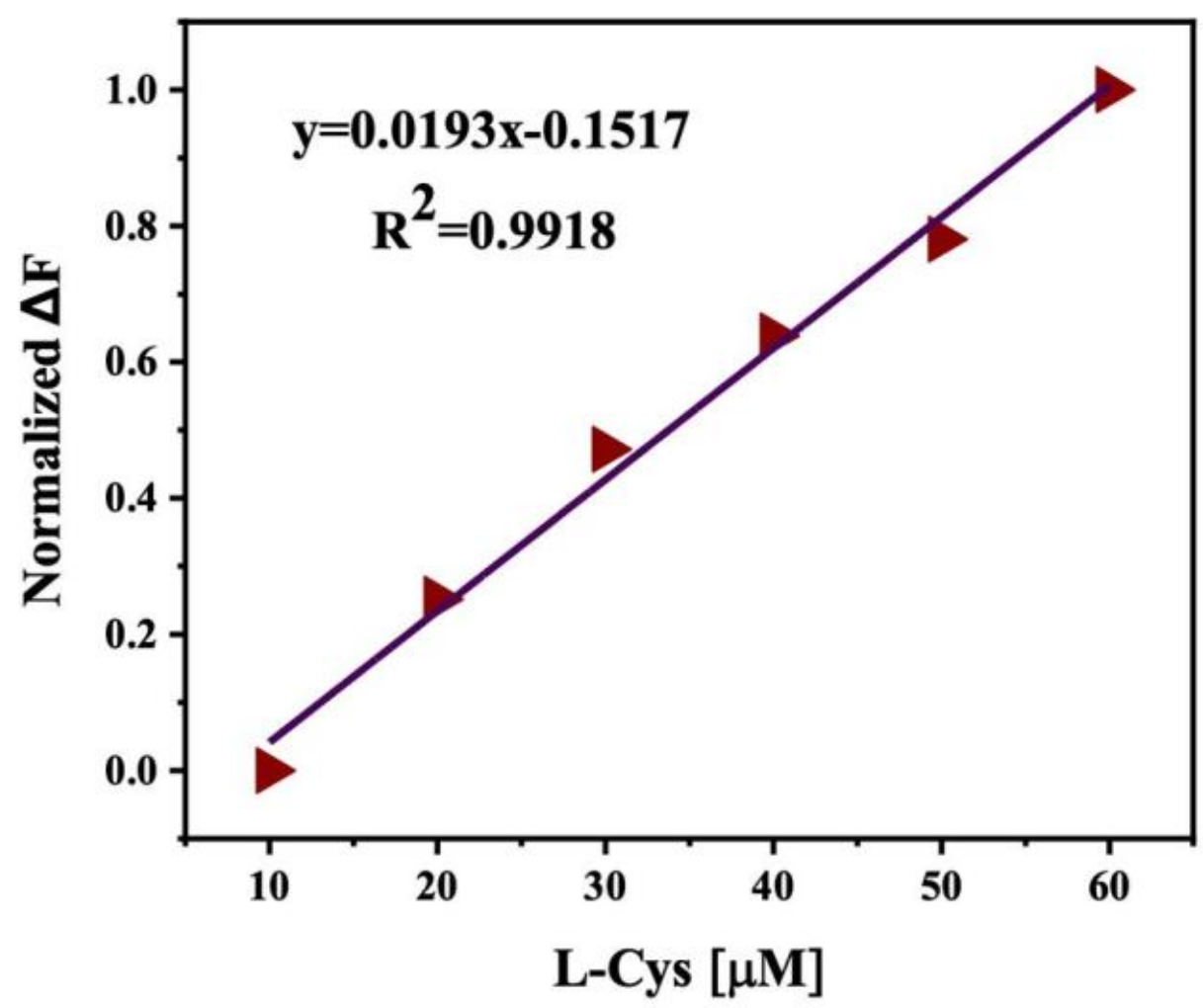

Fig. S9. Calibration curve showing the change in fluorescence produced by increasing concentrations of L-Cys within the linear response range in the presence of 10 µM **P3** in Britton-Robinson Buffer (pH 7): DMSO (95:5).

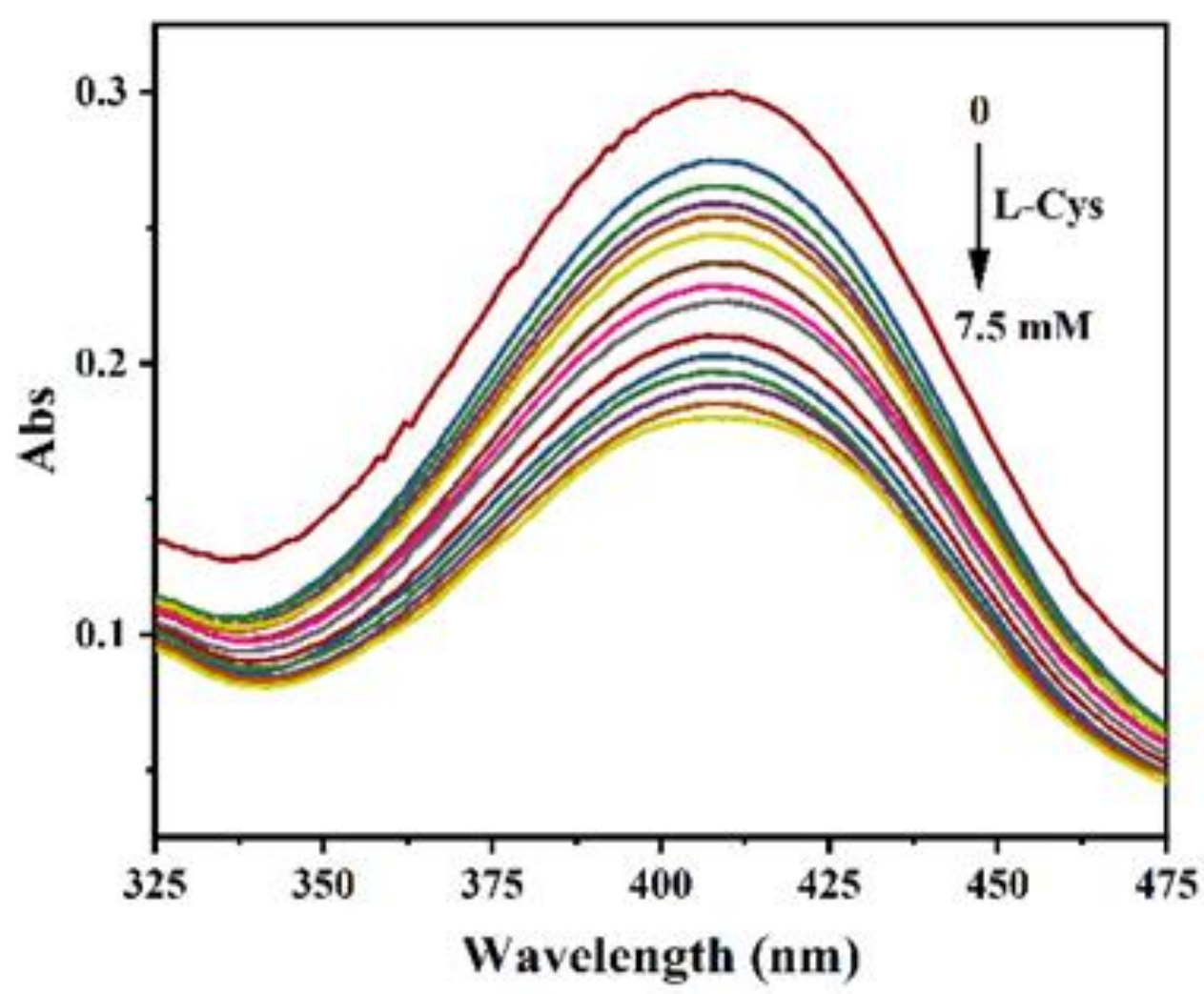

Fig. S10. UV absorbance spectral titration with L-Cys and 10 µM **P3** in Britton-Robinson Buffer (pH 7): DMSO (95:5).

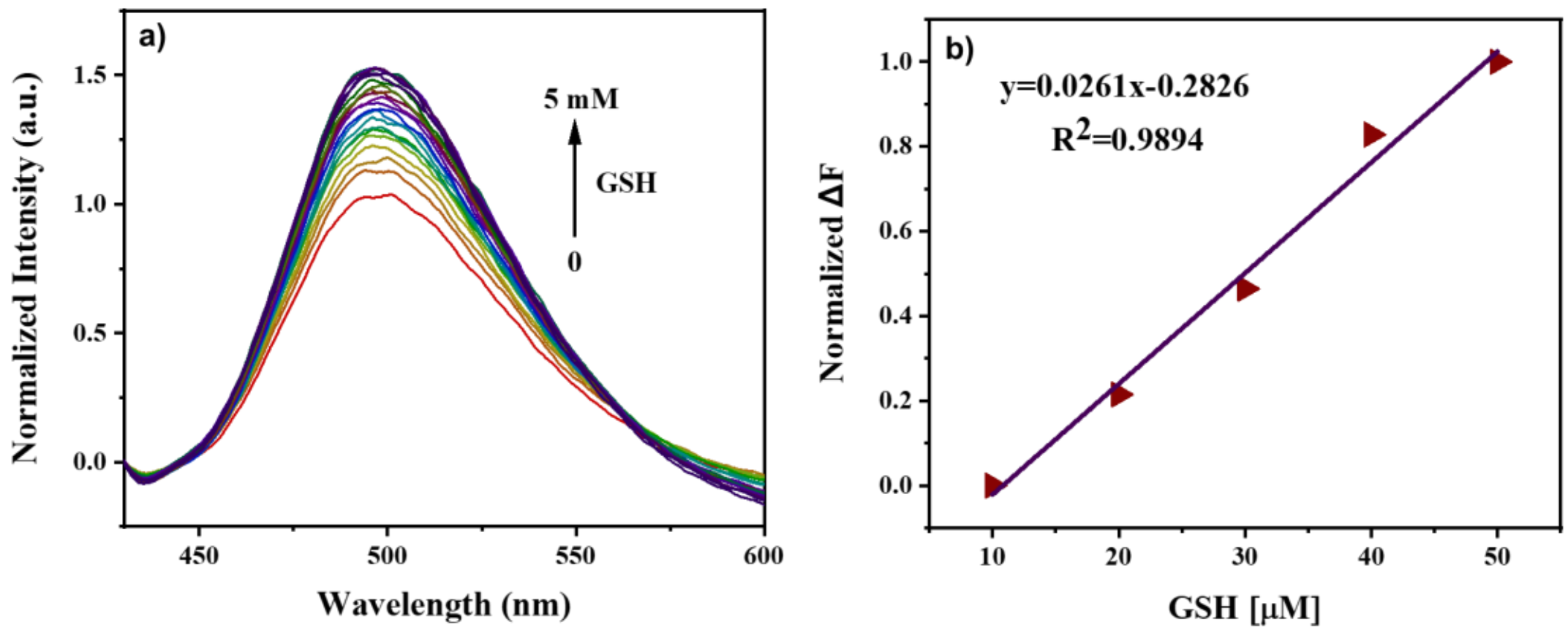

Fig. S11. Impact of GSH concentration on the fluorescence intensity of 10 µM solution of **P3** at pH 7: **a)** Normalized fluorescence emission spectra and **b)** calibration curve within the linear response range.

Table S1. Intraday and interday accuracy and precision studies (n = 3).

	**Concentration (µM)**	**Detected Concentration (µM)**	**Recovery (%)**	**RSD (%)**
**Intra-day**	30.00	29.58	98.60%	1.24
	40.00	40.26	100.66%	1.02
	50.00	50.51	101.02%	1.67
**Inter-day**	30.00	29.84	99.48%	1.64
	40.00	40.29	100.73%	1.20
	50.00	50.52	101.04%	1.67

Table S2. Statistical comparison of the results obtained using probe **P3** and the Ellman's reagent method as determined per the Student's t-test.

	Variable 1	***Variable 2***
**Mean**	87.4771	87.3881
**Variance**	0.0035	0.0298
**Observations**	15	15
**Pearson Correlation**	0.1547	
**Hypothesized Mean Difference**	0	
**df**	14	
**t Stat**	**1.989**	
**P(T<=t) one-tail**	0.0333	
**t Critical one-tail**	1.7613	
**P(T<=t) two-tail**	0.0666	
**t Critical two-tail**	**2.1447**	

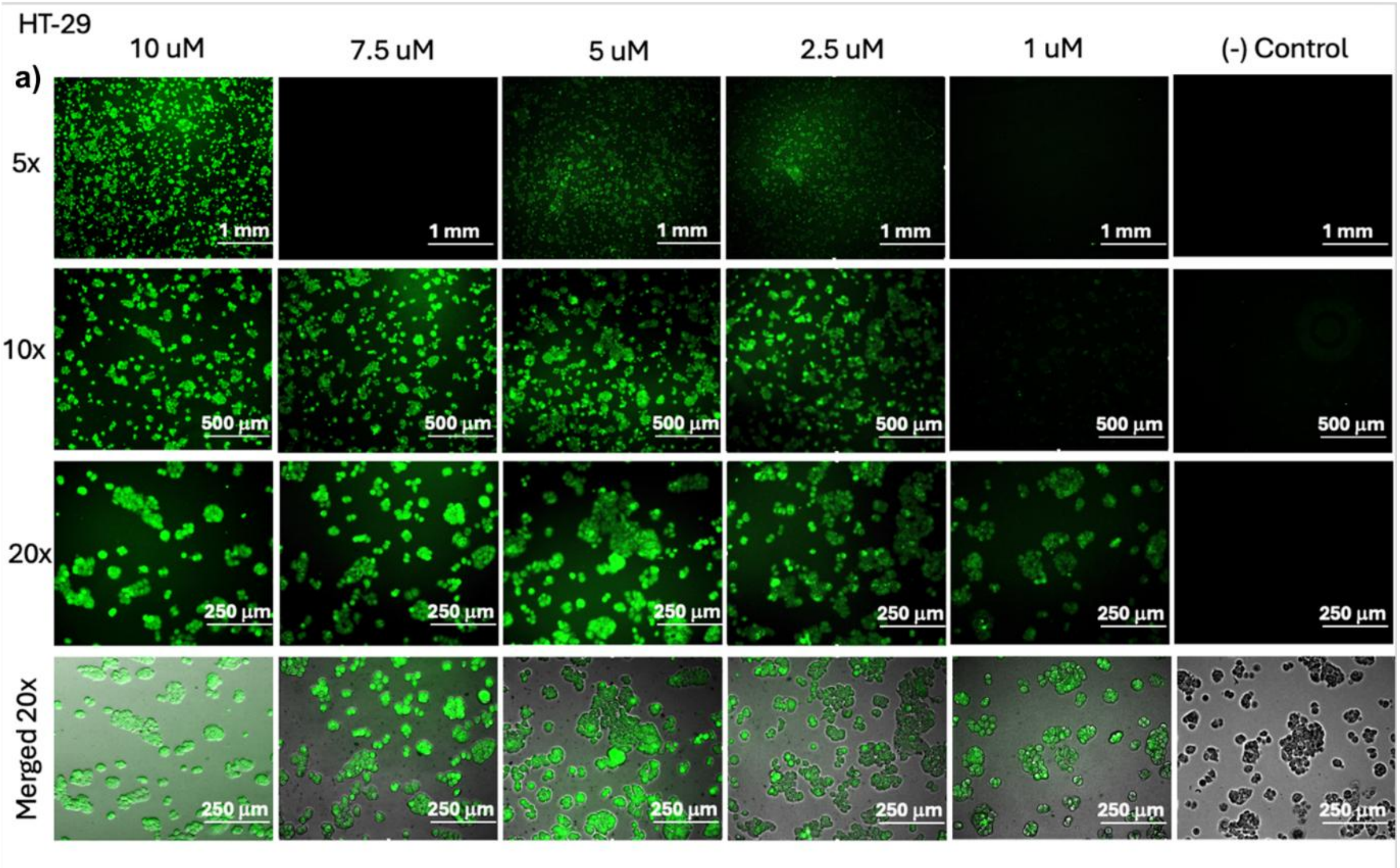
HT-29
a)
10 uM
7.5 uM
5 uM
2.5 uM
1 uM
(-) Control
5x
10x
20x
Merged 20x
1 mm
500 μm
250 μm

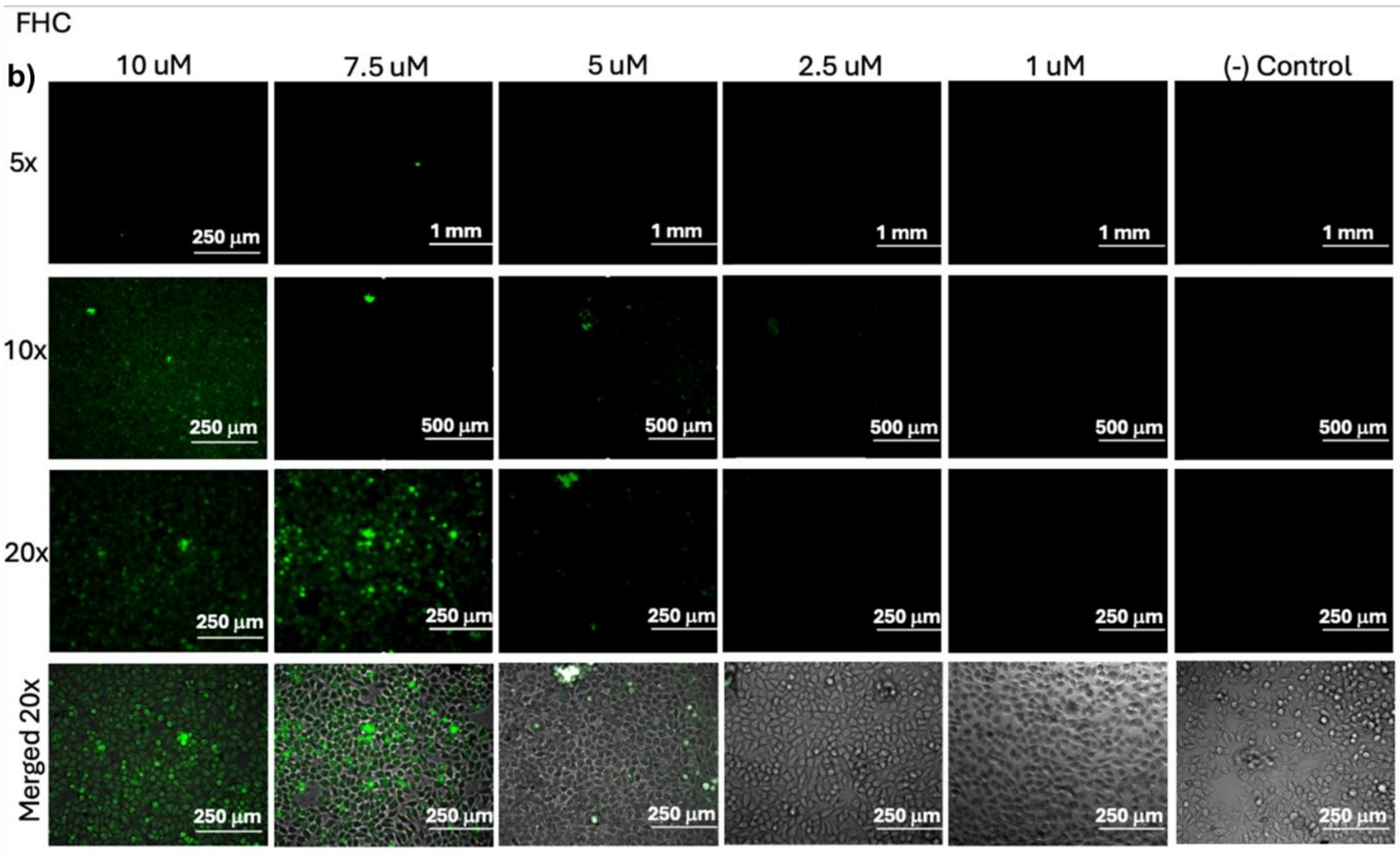
FHC
b)
10 uM
7.5 uM
5 uM
2.5 uM
1 uM
(-) Control
5x
10x
20x
Merged 20x
1 mm
500 μm
250 μm

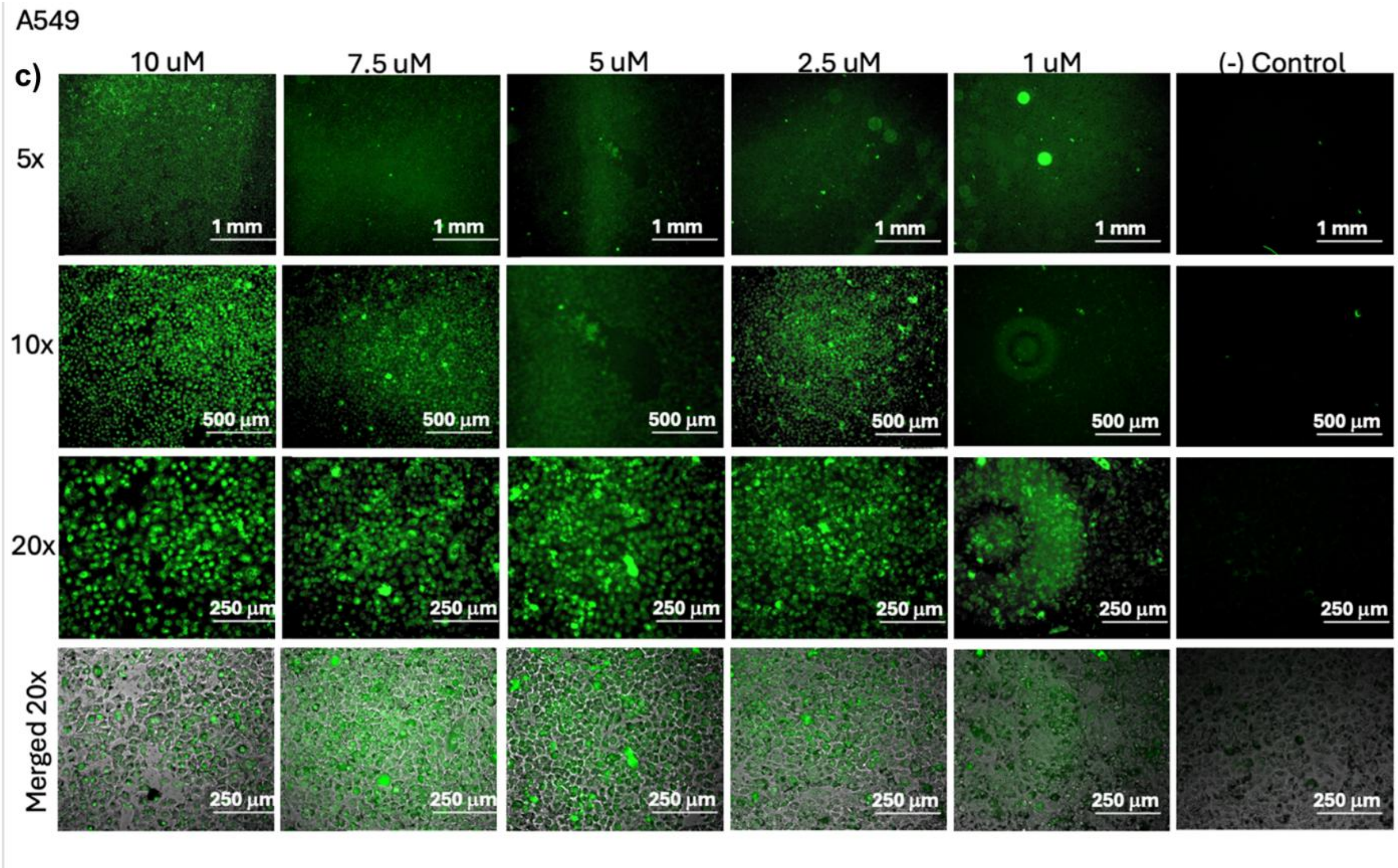

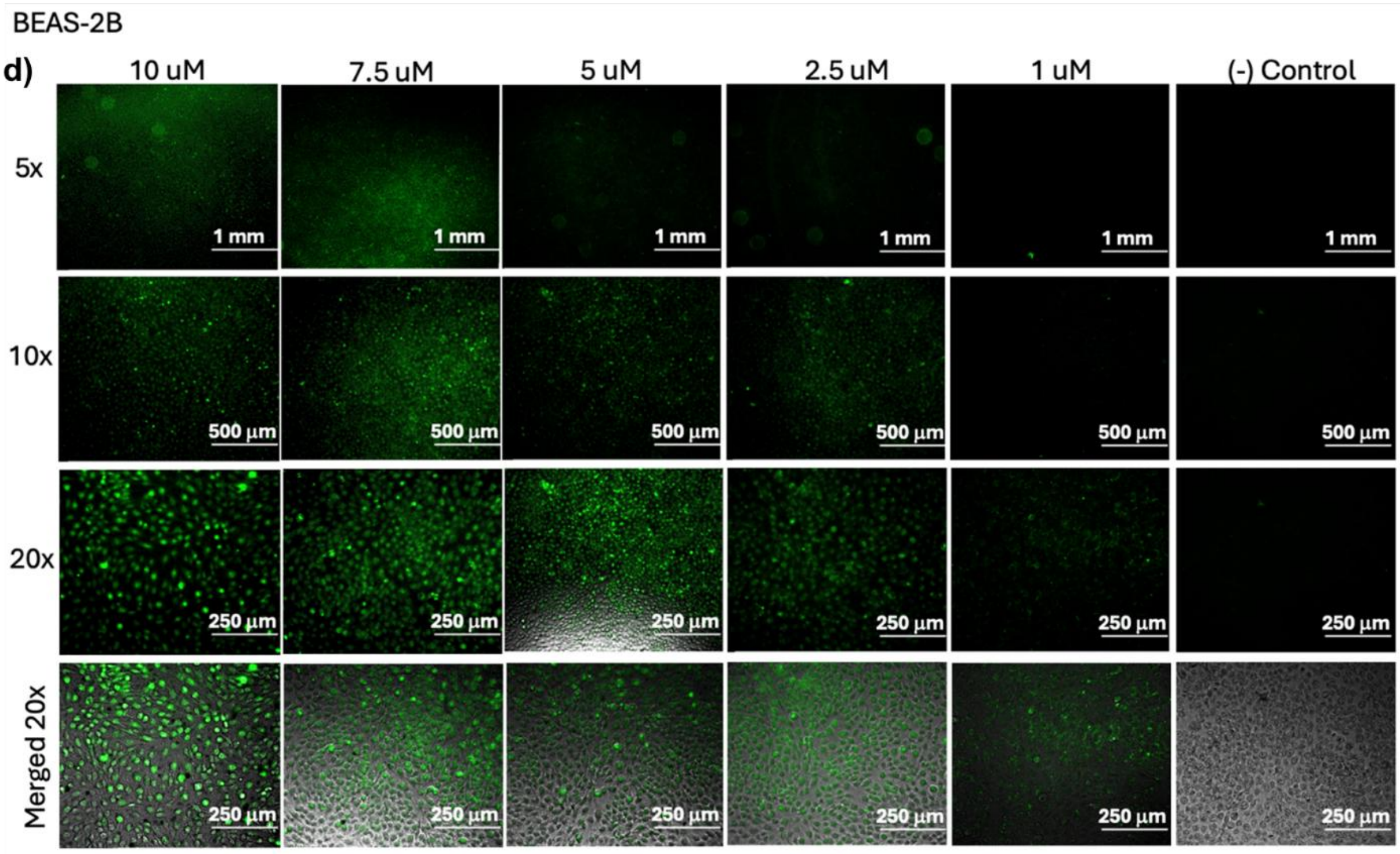

Fig. S12. Optimization of P3 concentration and microscope objective magnification for fluorescence cell imaging (a) Cancerous colon cells (HT-29) after treatment with **P3**, (b) healthy colon cells (FHC) after treatment with **P3**, (c) cancerous lung cells (A549) after treatment with **P3**, (d) healthy lung epithelial cells (BEAS-2B) after treatment with **P3**.

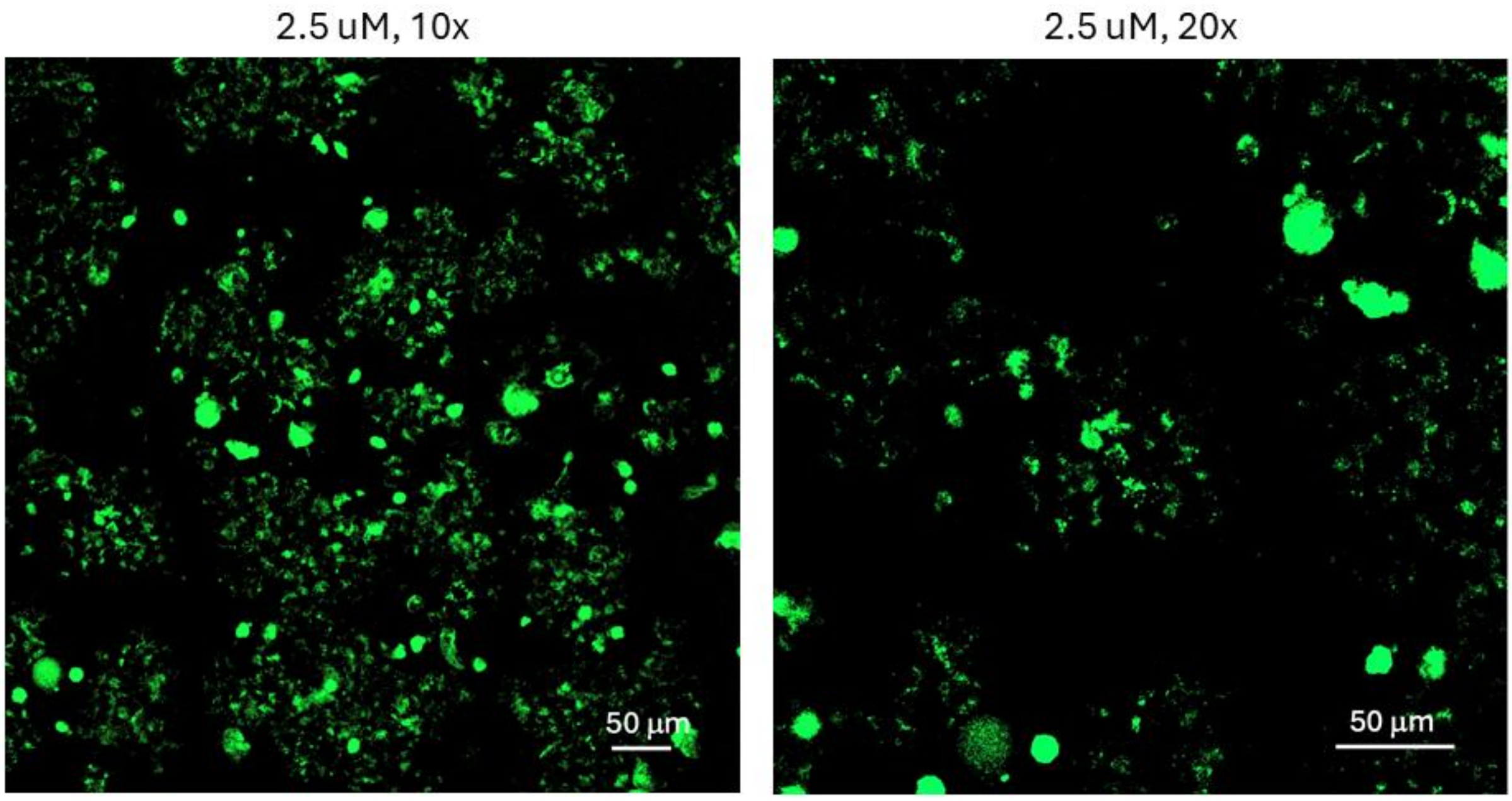

Fig. S13. Confocal fluorescence images of HT-29 cells stained with **P3** (2.5 µM, 10 min incubation) at 10× (left)and 20× (right) magnification; λ_{ex}= 488 nm, emission collected at 516 nm. Scale bars, 50 µm.

Table S3. Comparison of the sensing performance and practical applicability of P3 against existing literature.

**Target thiol**	**Fluorescence response**	**Solvent system**	**Stokes shift (nm)**	**Linear range**	**LOD**	**Application**	**Ref.**
Cys	Fluorescence quenching	HEPES buffer:ethanol (9:1 v/v), pH 7.16	91	0–40 µM	8 µM	-	1
Cys	Fluorescence quenching	PBS buffer, pH 7.4	125	0–100 µM	0.344 µM	Food samples and HeLa cell imaging	2
Cys, Hcy and GSH	Fluorescence enhancement	HEPES buffer: CH_3CN (1:4 v/v),	161	0.0–9.0 µM	0.8 nM, 1.1 nM, 0.6 nM	Cell imaging	3
GSH, Cys and Hcy	Fluorescence enhancement	Phosphate buffer, pH 7.4	20-30	0–40 µM	1.2 nM 1.9 nM 4.7 nM	Subcellular imaging	4
Cys, Hcy, GSH and H_2S	Fluorescence enhancement	PBS pH 7.4 with 20% EtOH	96	30–300 µM	0.42 µM 0.52 µM 0.38 µM 0.22 µM	Cell imaging	5
GSH, Cys and Hcy	Fluorescence enhancement	PBS:EtOH (1:1 v/v), pH 7.4	121	0–10 equiv	2.97 µM 2.08 µM 4.15 µM	HeLa and zebrafish embryo imaging	6
Cys and Hcy	Fluorescence enhancement	DMSO/HEPES buffer = 1:4 v/v, pH 7.4	148-203	0–40 µM	4.5 µM	live vero cell imaging	7
Cys and GSH	Fluorescence enhancement	DMSO/ BRT pH 7 = 1:20 v/v	90	10–60 µM 10–50 µM	5.32 µM 5.94 µM	Food supplement protein characterization and cancer cell detection	**This work**

References

1. P. Tao, D. Chen, Z. Xu, Y. Song, H. Li and C. Xian, Color. Technol., 2020, 136, 75–86.
2. W. Jiang, Q. Diao, W. Luo, L. Lv, T. Li, Q. Min, J. Bing and M. Zhang, Chemosensors, 2025, 13, 413.
3. X. Liu, L. Gao, L. Yang, L. Zou, W. Chen and X. Song, RSC Adv., 2015, 5, 18177–18182.
4. A. Mondal, N. Pareek, A. R. Khan, S. K. Sahoo, A. Samanta and S. Dutta, Analyst, 2025, 150, 4155–4168.
5. K. Xu, L. He, Y. Yang and W. Lin, New J. Chem., 2019, 43, 2865–2869.
6. J. Liao, S. Tang, R. Yang and Y. Han, Dyes Pigm., 2026, 251, 113726.
7. A. K. Mahapatra, S. Manna, P. Karmakar, K. Maiti, R. Maji, D. Mandal, R. Uddin and S. Mandal, Supramol. Chem., 2017, 29, 59–68.
